# Changing social composition of internet-use frequency categories and leisure exercise in China: a repeated cross-sectional study

**DOI:** 10.3389/fpubh.2026.1965960

**Published:** 2026-10-22

**Authors:** Wenfeng Zhu, Yi Lin, Banghui Hong, Jinfeng Liu

**Affiliations:** 1 School of Physical Education, Guizhou Normal University, Guiyang, China; 2 Graduate School of Human-Environment Studies, Kyushu University, Fukuoka, Japan

**Keywords:** China, digital inequality, internet-use frequency, leisure exercise, repeated cross-sectional surveys, social composition

## Abstract

**Background:**

As frequent internet use becomes widespread, the social population represented by nonfrequent internet users may change. We examined who these users were, how their composition differed across survey years, and whether their leisure-exercise differences from frequent internet users were similar in magnitude across years.

**Methods:**

Nine Chinese General Social Survey cross-sections from 2010–2023 provided 90,457 records for descriptive analyses of adults aged 18 years or older and 71,218 records for association analyses at ages 30–74. Survey-weighted models standardized exercise probabilities to each wave's covariate distribution. The outcome was leisure exercise daily or several times per week. Three- and five-category comparisons examined the binary grouping alongside composition and observed support.

**Results:**

Frequent internet users accounted for 19.15% of the adult descriptive sample in 2010 and 71.73% in 2023. Among nonfrequent internet users, never users comprised 82.82% in 2010 and 48.88% in 2023. Nonfrequent users' mean ages were 49.35 and 62.39 years, and mean personal-income percentiles were 45.75 and 37.62, respectively. Frequent-minus-nonfrequent exercise differences ranged from 2.12 to 10.09 percentage points; all nine point estimates were positive, with 95% intervals including zero in 2010 and 2023. Frequent-minus-never differences ranged from 4.29 to 13.65 points, versus −0.19 to 7.07 points for frequent-minus-low-frequency use. In the 2023 association sample aged 30–74, respondents aged 30–35 accounted for 14.43% of the weighted population, but none were never internet users; standardized comparisons involving never use therefore required age extrapolation.

**Conclusion:**

The same nonfrequent internet-use label encompassed different behavioral mixtures and social profiles across surveys. Interpreting leisure-exercise associations across years requires attention to the people represented by the frequency groups, the standardization population and observed support.

## 1 Introduction

Internet access has become widespread in China, with smartphones and online services increasingly embedded in daily life (1, 2). Official time-use reports describe concurrent increases in internet and exercise time and a decline in television viewing between 2008 and 2018 (2). Increasing use overall does not identify who moves from nonfrequent to frequent use or who continues to use the internet infrequently. If social groups move toward frequent use at different rates, their age, education and other characteristics within each frequency group may change even when the classification stays the same. We therefore ask: as frequent internet use changes from a minority practice to part of everyday life for the majority, do nonfrequent internet users still represent similar social populations, and how should leisure-exercise differences between these groups be interpreted across survey years?

We address these questions using nine repeated cross-sectional Chinese General Social Surveys from 2010 to 2023. We describe adults aged 18 years or older and estimate wave-specific exercise associations among respondents aged 30–74. To clarify the binary comparison (frequent versus nonfrequent use), we further distinguish never, low frequency and frequent internet users and examine all five original response levels.

## 2 Theoretical framework and research questions

### 2.1 Why internet diffusion may change the composition of user groups

Internet diffusion may change the composition of frequency groups because social groups adopt the internet at different times and to different extents. Diffusion theory examines how innovations spread through social groups over time, providing a starting point for understanding this uneven process (3). Chinese longitudinal evidence found widening age gaps in perceived internet importance and access among less-educated people, but narrowing gaps among more-educated people (4). Surveys in Great Britain and Sweden found that internet non-users became more concentrated in disadvantaged groups and that their reported reasons for non-use changed (5). The former shows that diffusion does not imply parallel convergence across age and education groups; the latter shows that the social profile associated with non-use can itself change over time.

The nonfrequent internet users examined here form a broader group than internet non-users: our category combines never, rarely and sometimes responses. Even with an unchanged binary rule, the proportions of these three responses may differ, and the social profiles of their respondents may also change. We therefore examine both the internal frequency mixture and the social composition of users to establish whom the same label represents in each year.

Understanding these group differences also requires distinguishing access to the internet from how it is used. Van Dijk distinguishes motivational access, physical access, digital skills and actual use as four stages of access to and use of digital technology (6). Physical access means having usable devices and a network connection; these conditions alone do not imply willingness, proficiency or frequent use. Helsper's corresponding fields model links digital engagement to economic, cultural, social and personal resources (7). These frameworks place use frequency in the context of people's resources, abilities and living conditions.

This distinction also helps explain heterogeneity within nonfrequent use. Internet non-use may reflect the fit between technology and people's circumstances (8); low-frequency users and non-users may differ in social characteristics and motivations (9), while more internet use does not necessarily mean more beneficial online activities (10). We therefore describe age, education, hukou and personal income separately rather than treating nonfrequent use as a uniform form of social disadvantage. Chinese evidence that the association between education and digital technology use differs across hukou groups also supports examining several dimensions of user composition (11).

Group composition and participation within a social group answer different questions. For example, the proportion of frequent users who have tertiary education describes the educational composition of frequent users; the proportion of tertiary-educated adults who use frequently describes participation within that educational group. The former uses the total weight of frequent users as its denominator, and the latter the total weight of tertiary-educated adults. These proportions can move in different directions as use increases in other educational groups. We report both proportions alongside contemporaneous population characteristics to distinguish changes in the composition of a use group from changes in participation within a social group.

### 2.2 Why internet use and leisure exercise may be associated

Health-lifestyle theory relates health practices to agency and social structure (12). Education, economic resources, age and institutional position may shape both internet use and opportunities for leisure exercise. Chinese surveillance records social differences in leisure activity (13), while community evidence shows that socioeconomic gradients differ between exercise, occupational and domestic activity (14). Exercise differences between internet-use groups may therefore reflect shared social conditions as well as relationships between the activities themselves.

Beyond shared social conditions, internet activities may relate to exercise through daily routines: online information may help people find exercise opportunities and online communication may help them arrange activities together, while some online activities may compete for exercise time. Chinese studies have examined internet access and duration of use (15), access through phones or computers in relation to exercise frequency (16), and internet frequency and purpose among older adults (17). In deprived Glasgow communities, internet access was associated with lower odds of low physical activity, measured using the short International Physical Activity Questionnaire (18). These studies motivate examining links between internet use and physical activity. Here, we focus on internet-frequency groups and leisure exercise, examining the magnitude of their association across survey years.

### 2.3 What matters when comparing exercise associations across years

Interpreting exercise associations across years first requires identifying the people included on each side of a comparison. If never and low-frequency users differ in composition or exercise, a single contrast with the combined group cannot show which component accounts for the comparison. We therefore first compare frequent with nonfrequent users, then separate never from low-frequency users, and use five-category analyses to examine differences between adjacent responses.

Comparing exercise probabilities also requires a common population composition for the use groups. We use all respondents aged 30–74 included in each year's association analysis as that year's reference population, also termed the standardization target. For example, to compare frequent and nonfrequent use, we retain these respondents' age, education and other characteristics, predict exercise under each of the two use frequencies, and average the predictions using survey weights (19, 20). The resulting difference thus compares model predictions for the same population characteristics rather than directly subtracting the groups' observed exercise proportions. Because the reference population varies with the sample across years, cross-year interpretation also requires considering population composition. Predictions rely on model extrapolation where a use group has no respondents with ages or social characteristics found in the reference population.

### 2.4 Three research questions

The framework leads to three research questions:

1. Who are nonfrequent internet users? How do their age, education, hukou and personal income compare with frequent users and the population surveyed in the same year?
2. How does this social composition differ across survey years? How do nonfrequent users' characteristics and their mixture of never, rarely and sometimes use vary across surveys, relative to frequent users and the contemporaneous population?
3. Are exercise associations similar in magnitude across survey years? What is the standardized leisure-exercise probability difference between frequent and nonfrequent internet users in each wave, and what does separating never from low frequency use reveal?

## 3 Data and analytical methods

### 3.1 Survey years and study design

The Chinese General Social Survey began in 2003; Bian and Li describe its early sampling and fieldwork design (21). We analyzed nine public-use repeated cross-sections: 2010, 2011, 2012, 2013, 2015, 2017, 2018, 2021 and 2023. The analysis used in-person interviews and excluded the 2023 telephone branch because its routing differed (Supplementary Table S1).

### 3.2 Defining descriptive and association samples

D18 comprised in-person respondents aged 18–110 with valid internet-use responses and positive finite official primary weights, irrespective of exercise or social-profile completeness. Age was survey year minus birth year. D30 restricted D18 to ages 30–74; A30 additionally required valid exercise, sex, ethnicity, education, hukou and province. C30 required complete marital status, employment and household income for extended adjustment (Supplementary Table S2).

Association analyses used ages 30–74 to reduce sparse use groups at the age extremes: the in-person samples contained only 13 frequent users aged 75 or older in 2010 and 16 nonfrequent users aged 18–29 in 2023. D30 used the same age range to align descriptive results with association analyses; observed support for each internet-use classification was still assessed within A30. Additional D18 and D30 descriptions were restricted to the ten provinces observed in all nine waves to examine composition within a common geographic scope.

### 3.3 Measuring internet-use frequency, leisure exercise and social characteristics

Internet-use frequency was measured by the internet item in the past-year media-use battery (A28), which also covered newspapers, magazines, radio and television. Responses were never, rarely, sometimes, often, and always or very frequently, with the top label varying by wave. Frequent internet use comprised responses 4–5 and nonfrequent use 1–3. Three-category analysis distinguished never (1), low frequency (2–3) and frequent use (4–5); five-category models treated the original responses as an unordered factor (Supplementary Table S1).

The outcome was leisure-exercise frequency during the past year. Responses 1–2 (daily or several times per week) were contrasted with responses 3–5 (several times per month, several times per year or less, and never). The questionnaire had no separate once-per-week option. Special nonresponse and inapplicable codes were treated as missing.

We reported sample sizes and weighted internet-use frequency distributions by survey year and descriptive sample. Internal composition described responses 1–3 among nonfrequent internet users and 4–5 among frequent users. Social profiles covered age, education, agricultural versus other hukou and personal-income rank, with variable-specific observed denominators. Both conditional distributions defined in Section 2.1 and contemporaneous population profiles are reported in Supplementary Tables S3–S5.

Personal-income percentiles describe respondents' relative positions in that year's adult income distribution and used the weighted mid-distribution rank among D18 respondents with observed personal income in each wave, retaining ties and a common reference across groups and sub-samples. Extended adjustment instead used household-income ranks calculated within each wave's complete C30 sample.

### 3.4 Estimating and comparing exercise probabilities across internet-use groups

To compare exercise probabilities for different internet-use groups in a common population within each year, we fitted survey-weighted logistic models and then standardized their predictions. For every A30 respondent, we retained observed age, education and other characteristics, set internet-use frequency in turn to each comparison group, predicted exercise probability and averaged those predictions using that year's survey weights (19, 20). Separate binary, three-category and five-category models were fitted within each year using the R survey package (22). Core adjustment included an age natural spline with 4 degrees of freedom, sex, Han versus other ethnicity, five-category education, agricultural versus other hukou and province fixed effects, without interactions between internet-use frequency and other variables. The standardized probability for group k in wave t was:


$$\begin{array}{l} {\hat{p}}_{tk}\;=\;\underset{i\in {A30}_{t}}{\sum }{\tilde{w}}_{ti}{\hat{m}}_{t}\left(k,C_{ti}\right),\;{\tilde{w}}_{ti}\;=\;\frac{w_{ti}}{\sum_{j\in {A30}_{t}}w_{tj}} \end{array}$$ (1)

Here, ${\hat{m}}_{t}$(k, *C*_*ti*_) is the fitted exercise probability with internet-use group set to k and respondent i's observed covariates retained. Standardized probability differences were reported in percentage points. Binary models compared frequent with nonfrequent use; three-category models compared frequent with never, low frequency with never, and frequent with low frequency use. For five-category models, we reported all five standardized probabilities and the four adjacent-category differences (2−1, 3−2, 4−3 and 5−4) in each wave. For the binary and three-category contrasts, we also reported the ratio of the two standardized exercise probabilities, with the first named group as numerator. These dimensionless probability ratios describe cross-sectional associations.

Official primary weights were weight for 2010–2012, weight_raking for 2013–2021 and weight1 for the 2023 in-person branch. Because verified official primary sampling units and strata were unavailable across releases, province served as the geographic clustering proxy. Joint influence-function calculations included coefficient uncertainty, variability in the empirical target and their covariance; contrasts used the full covariance matrix of standardized probabilities. Probability intervals used a logit transformation, whereas difference intervals used the probability scale. Probability-ratio intervals used the log ratio and the same full covariance matrix, with t critical values at G−1 degrees of freedom, where G was the number of provinces in that wave's A30 sample.

Pointwise intervals used a t approximation with degrees of freedom equal to the number of provinces minus one (15–30), specified independently of svyglm residual degrees of freedom. This province-proxy inference was used consistently across the wave-specific comparisons (Supplementary Tables S6–S9, S13).

### 3.5 Observed coverage of comparison groups and adjustment on the same sample

The target population comprises all respondents aged 30–74 in each year's main association sample (A30), with composition described using that year's survey weights. Observed support assesses whether each internet-use group contains respondents with the ages and social characteristics found in this target population. We reported group counts, effective sample sizes and age ranges, and calculated the weighted proportions of all A30 respondents outside each group's observed ages or at unobserved covariate levels. Cross-tabulations used 5-year age bands (30–34, 35–39, and so forth), sex, ethnicity, education, hukou and province. Joint age-band, sex, education and hukou groups further identified combinations with no respondents in a given internet-use group, revealing reliance on extrapolation not apparent from examining characteristics separately (23). Model convergence, rank and prediction diagnostics were also reported (Supplementary Table S9).

For the binary comparison, unadjusted (E0), core-adjusted (E1) and extended-adjusted (E2) models used identical C30 records, weights and within-wave standardization targets. E2 added marital status, employment and household-income rank. Comparison with A30 E0 and E1 estimates separated numerical changes associated with sample restriction from those associated with additional adjustment (Supplementary Table S10).

### 3.6 Auxiliary summaries and sensitivity analyses

Auxiliary equal-wave summaries averaged the differences across the nine surveys, giving each year equal weight, assuming zero cross-wave covariance; their variance was the sum of wave variances divided by the squared number of waves. Identical-record adjustment summaries used Satterthwaite t intervals; other analyses specified normal-reference or t intervals (Supplementary Tables S10, S11).

Auxiliary analyses examined alternative age ranges, weights, exposure definitions and exercise-frequency cutoffs, as well as interview mode. A separate 36-specification analysis varied link functions, age forms, education coding and internet-by-sex interactions, yielding 324 wave fits on common within-wave samples and targets (24). We omitted each survey year in turn and recalculated the average association over the remaining 8 years. We also removed each province across all years in which it appeared to assess whether individual years or provinces drove the summary association. Separately, we calculated confidence intervals using random multipliers applied to province-level contributions and by refitting models after deleting one province from one wave at a time, to assess sensitivity of interval estimates to the inference method (Supplementary Tables S11–S13). For descriptive weight comparisons, we held D18 records fixed and compared primary weights with equal record weights, primary weights capped at the wave-specific 1st and 99th percentiles, and the released weight field in the five raking waves. D30 comparisons used the same D18-derived cap thresholds. Paired province-cluster linearization retained covariance between weight schemes (Supplementary Table S3D).

Extended-adjustment sensitivity analysis used chained-equation multiple imputation (25): 50 datasets, ten iterations, predictive mean matching for income, and categorical models for marital status and employment. Exposure, outcome and structural non-asking were not imputed. Standardized differences were pooled using Rubin's rules with finite-sample degrees of freedom. The identical-sample comparison used complete C30 records. An additional sensitivity analysis added self-rated health to the extended adjustment set (Supplementary Table S10).

We additionally applied the E-value formula to the nine E1 binary standardized probability ratios to obtain necessary sensitivity thresholds under the strong null that the mean causal risk ratio equals one within every stratum of measured covariates (Supplementary Table S6B). This calculation imposes uniform upper bounds on the conditional associations of unmeasured factors with Internet use and exercise and retains the existing probability-ratio confidence intervals. All analyses used R 4.6.1; survey-weighted models used survey 4.5.

## 4 Results

### 4.1 Sample sizes and internet-use frequency distributions by year

Across the nine surveys, the descriptive sample of adults aged 18 years or older included 90,457 records (D18). Restriction to ages 30–74 yielded 71,512 records (D30), of which 71,218 had valid exercise and core covariates for association analysis (A30). Extended complete-case analyses included 64,000 records (C30). Wave-specific counts and the full exclusion flow are provided in Table 1 and Supplementary Table S2.

**Table 1:** Sample sizes and three-category counts.

Year	D18	D30	A30	C30	A30 Never	A30 Low	A30 Frequent
2010	11,708	9,376	9,307	8,327	6,707	1,190	1,410
2011	5,593	4,461	4,437	4,018	3,208	590	639
2012	11,753	9,370	9,345	8,427	6,226	1,338	1,781
2013	11,413	9,013	8,977	7,907	5,641	1,522	1,814
2015	10,917	8,478	8,424	7,798	4,873	1,367	2,184
2017	12,574	9,904	9,878	9,082	4,381	1,453	4,044
2018	12,780	10,094	10,066	9,125	3,872	1,768	4,426
2021	8,141	6,288	6,263	5,129	1,707	1,020	3,536
2023	5,578	4,528	4,521	4,187	768	870	2,883
Total	90,457	71,512	71,218	64,000	37,383	11,118	22,717

Counts are unweighted respondent records. D18: age ≥18, valid internet-use response and positive finite weight; D30: ages 30–74 within D18; A30: D30 with valid exercise outcome and complete core covariates; C30: complete extended-adjustment sample. These are repeated cross-sections, not follow-up counts of unique people.

In the adult descriptive sample, the weighted proportions reporting never, low frequency and frequent internet use were 66.96%, 13.89% and 19.15% in 2010, compared with 13.82%, 14.45% and 71.73% in 2023. The sequence was not strictly monotonic: frequent use reached 75.47% and never use 11.35% in 2021. Among adults aged 30–74 in D30, frequent use accounted for 13.66% in 2010 and 70.95% in 2023 (Figure 1, Supplementary Table S3). On the same D18 records, unweighted frequent-use percentages were 58.00% in 2021 and 62.84% in 2023, reversing the direction of the primary-weighted change. Across all nine waves, capping changed frequent-use percentages by −0.93 to 0.02 points; in the five raking waves, the alternative weight field gave percentages 4.73–8.82 points lower (Supplementary Table S3D).

**Figure 1:**
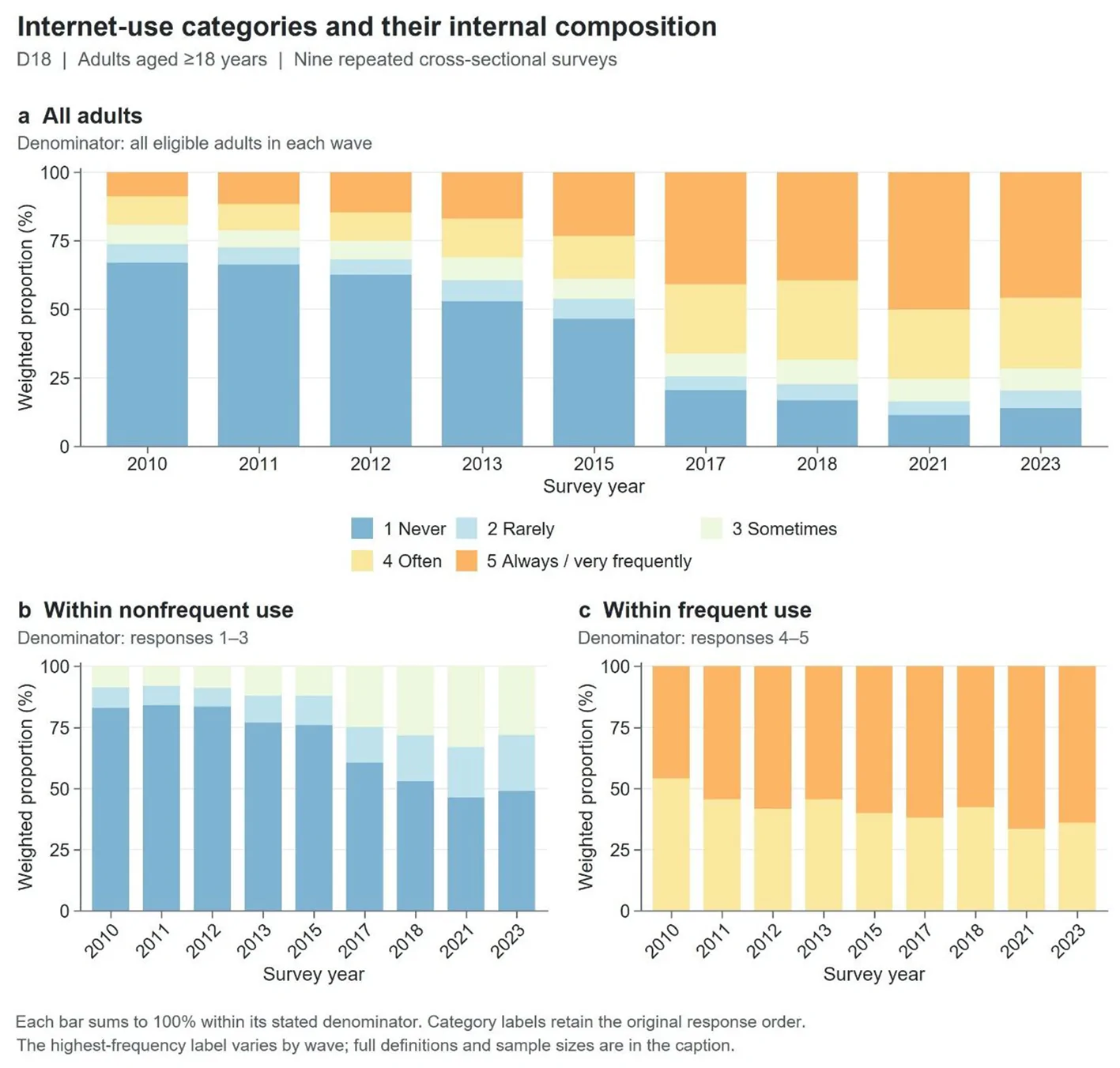
Internet-use frequency distributions and the composition of the binary categories across nine CGSS surveys. Survey-weighted percentages show **(a)** the five original internet-use responses in each year's adult descriptive sample (D18), **(b)** never, rarely and sometimes use among nonfrequent internet users, and **(c)** often and always/very frequently use among frequent internet users. The past-year media-use item defines nonfrequent use as responses 1–3 and frequent use as responses 4–5; the highest response label varies by year. D18 includes 90,457 in-person records aged at least 18 years with valid internet-use responses and positive finite official primary weights. Percentages sum to 100% within each year and panel, apart from rounding. Year-specific unweighted counts are in Table 1.

Among nonfrequent internet users in D18, never, rarely and sometimes responses accounted for 82.82%, 8.38% and 8.80% in 2010 and 48.88%, 22.97% and 28.15% in 2023. Among frequent internet users, the highest internet-frequency response accounted for 45.86% in 2010 and 64.01% in 2023. The recent internal-mixture change also depended on weighting: never users constituted 46.30% and 48.88% of D18 nonfrequent users under primary weights in 2021 and 2023, compared with 67.12% and 51.71% without weighting. These are descriptive comparisons, not tests of change between waves (Supplementary Table S3D).

The changing mixture also appeared within narrower age and geographic scopes. Never users comprised 85.92% and 44.00% of D30 nonfrequent users in 2010 and 2023. In the ten provinces observed in every wave, the corresponding endpoint shares were 84.15% and 49.25% for D18, and 87.47% and 43.78% for D30 (Supplementary Table S3).

### 4.2 Social characteristics of internet-use groups and differences across years

Nonfrequent internet users had different social profiles at the two endpoints. In 2010 and 2023, their weighted mean ages were 49.35 and 62.39 years, compared with 33.89 and 43.31 among frequent users and 46.39 and 48.71 in the D18 population. Tertiary education accounted for 5.11% and 3.93% of nonfrequent users, 49.59% and 29.85% of frequent users, and 13.63% and 22.52% of the population (Figure 2, Supplementary Table S4).

**Figure 2:**
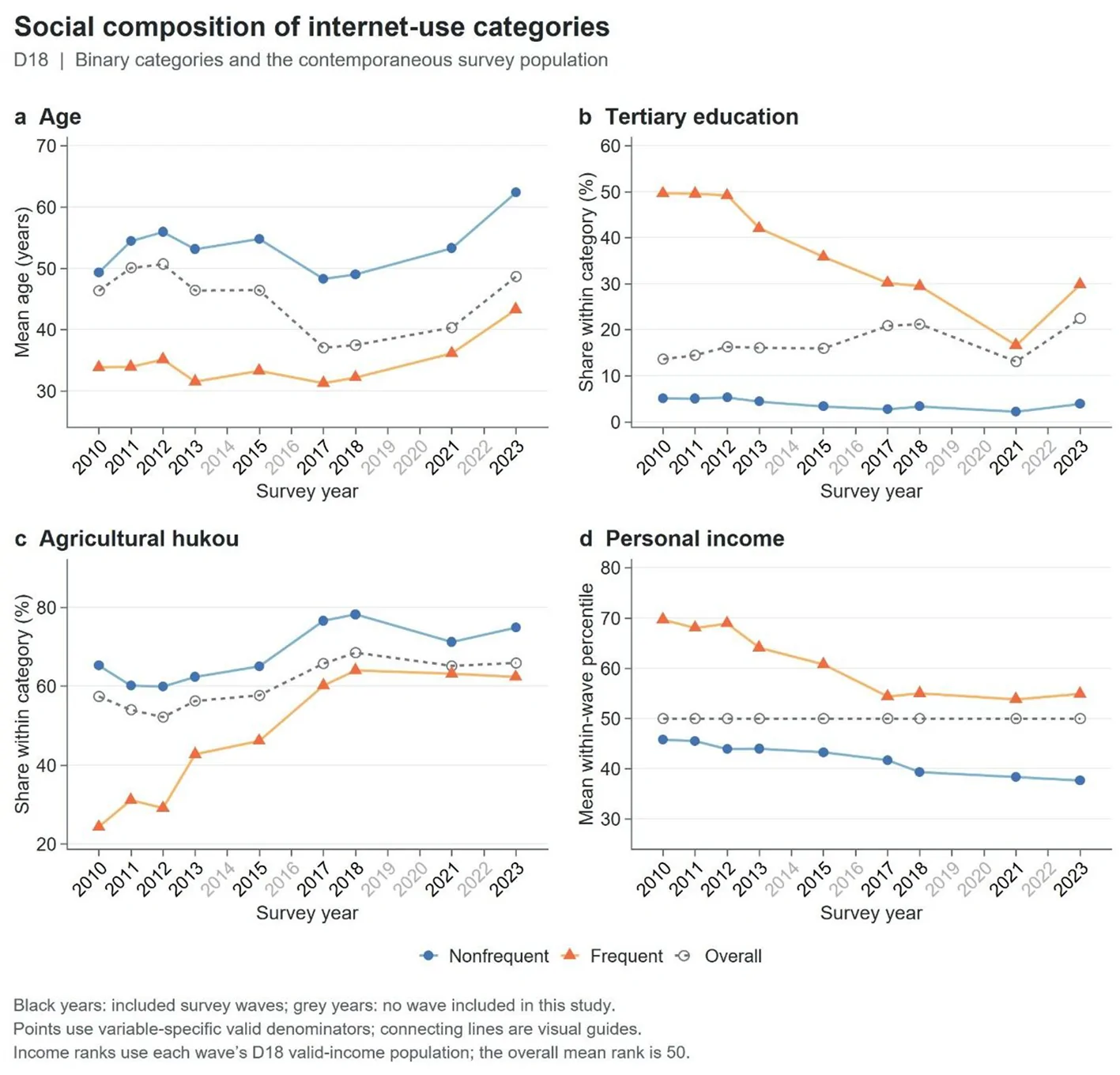
Social profiles of nonfrequent and frequent internet users relative to the contemporaneous adult sample. Survey-weighted summaries compare nonfrequent internet users (responses 1–3), frequent internet users (responses 4–5) and the overall adult sample (D18): **(a)** mean age in years, **(b)** tertiary education percentage, **(c)** agricultural hukou percentage and **(d)** mean personal-income percentile. Each summary uses respondents with valid observations for that variable within the indicated group and year. Income percentiles use the weighted cumulative-distribution midpoint ranks among D18 respondents with valid income, preserving ties and applying the same reference distribution to both groups. On the 0–100 scale, the overall mean is 50 by construction. Black year labels identify included survey waves; light-gray labels identify intervening years without an included wave. Points represent observed waves; connecting lines aid comparison. D18 includes 90,457 records aged at least 18 years; variable-specific denominators and missingness are in Supplementary Table S4.

Agricultural hukou accounted for 65.22% of nonfrequent internet users in 2010 and 74.80% in 2023, compared with 24.30% and 62.35% among frequent users and 57.39% and 65.87% overall. Mean personal-income percentiles were 45.75 and 37.62 among nonfrequent users and 69.68 and 54.89 among frequent users; the D18 reference mean was 50 by construction. Nonfrequent users were older and occupied a lower relative income position in 2023 than in 2010, while gaps between the groups varied across social dimensions.

Separating never from low frequency internet use revealed differences within the nonfrequent group. Mean ages among never and low frequency users were 51.51 and 38.91 years in 2010, and 67.72 and 57.30 in 2023. The never-versus-frequent mean-age gap was 17.62 years in 2010 and 24.41 in 2023.

Among never internet users, tertiary education accounted for 2.31% in 2010 and 1.67% in 2023; agricultural hukou accounted for 70.21% and 78.38%, and mean income percentiles were 42.88 and 33.46. Their absolute gaps from frequent users narrowed for education, hukou and income but widened for age. Low frequency users lay between never and frequent users on all four indicators at both endpoints (Supplementary Table S4).

The preceding results describe who makes up each use group. Changing the denominator shows how frequently people use the internet within each social group. Among adults aged 60–74, frequent internet use accounted for 2.73% in 2010 and 43.91% in 2023, while never use accounted for 90.89% and 33.04%. Among tertiary-educated adults, frequent use increased from 69.70% to 95.07%, alongside their declining share among frequent users (Supplementary Table S5).

Mean personal-income percentile among low frequency users was 60.16 in 2010 and 41.61 in 2023, compared with the D18 reference mean of 50 in each wave. D30 endpoint comparisons likewise showed a larger age difference and smaller absolute differences in tertiary education, agricultural hukou and personal-income percentile between never and frequent internet users (Supplementary Table S4).

### 4.3 Year-specific exercise differences across internet-use groups

In A30, frequent-minus-nonfrequent standardized exercise differences were 2.12 percentage points in 2010 (95% CI −0.49–4.73), 6.51 in 2011 (2.72–10.30), 5.78 in 2012 (2.84–8.72), 4.04 in 2013 (1.04–7.04), and 6.85 in 2015 (1.86–11.84) (Figure 3a, Supplementary Table S6).

**Figure 3:**
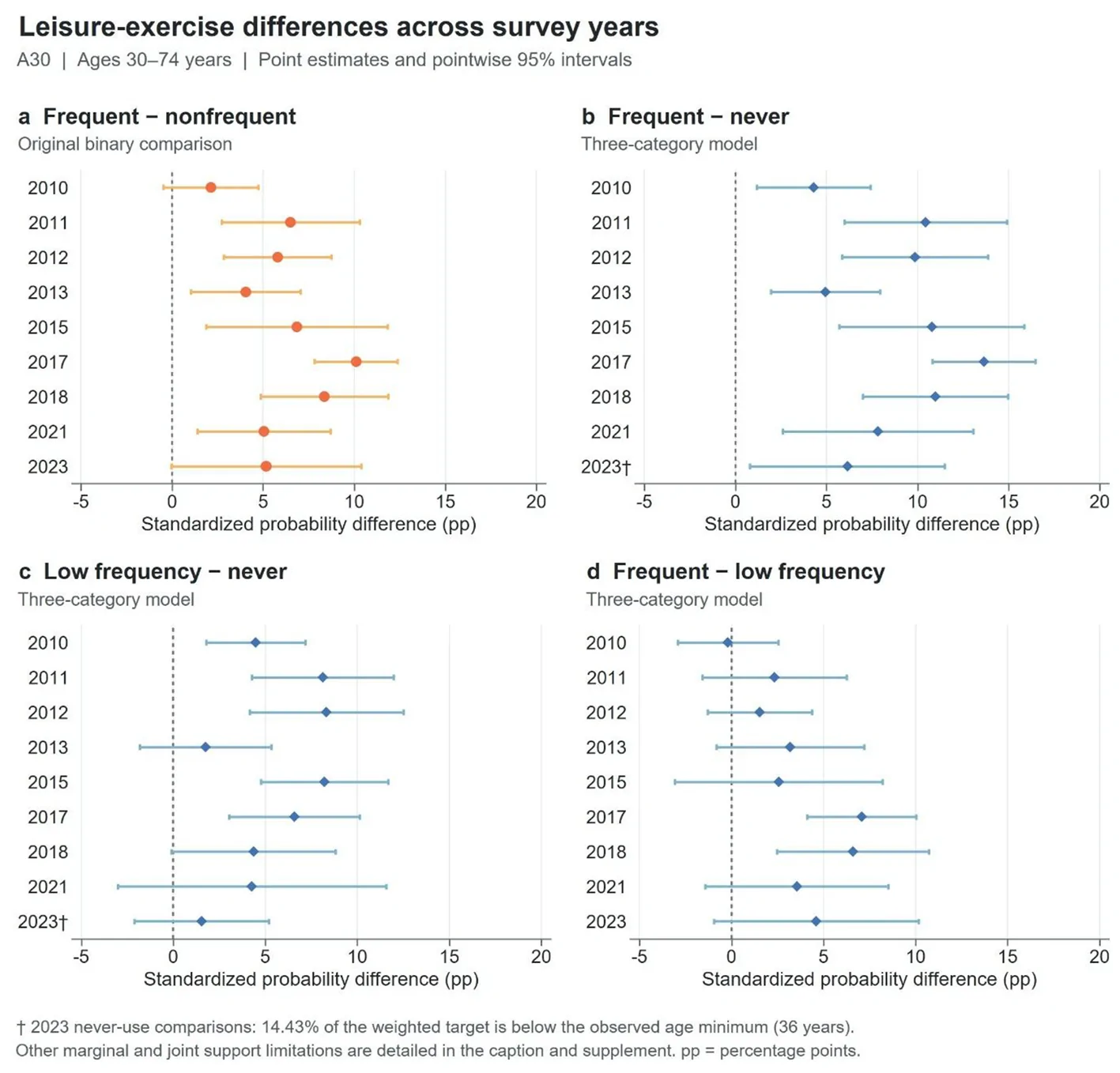
Wave-specific standardized differences in leisure exercise under binary and three-category definitions of internet use. Points and horizontal bars show standardized probability differences and pointwise 95% confidence intervals, in percentage points: **(a)** frequent minus nonfrequent internet use from the binary model; **(b)** frequent minus never, **(c)** low frequency minus never and **(d)** frequent minus low frequency use from the three-category model. Never, low frequency and frequent use correspond to responses 1, 2–3 and 4–5; nonfrequent use combines responses 1–3. The outcome is leisure exercise daily or several times per week. Separate survey-weighted logistic models use 71,218 A30 records aged 30–74 years and adjust for age (a natural spline with four degrees of freedom), sex, ethnicity, five-category education, agricultural hukou and province fixed effects. Predictions are averaged over each year's weighted A30 population. Intervals include model and empirical-target uncertainty and their covariance, using province clustering and a t approximation with the number of provinces minus one degrees of freedom (15–30). † In 2023, all A30 respondents were aged 30–74; ages 30–35 accounted for 14.43% of the weighted population, whereas observed never internet users were aged 36–74. Predicting never-use probabilities for ages 30–35 in **(b)** and **(c)** therefore required age extrapolation. Other marginal gaps were no frequent internet users in Tibet in 2010 (0.20% of the weighted target; a, b and d), an upper observed age of 73 among frequent internet users in 2011 (1.55% outside the range; a, b and d), and a lower observed age of 31 among never internet users in 2021 (3.13% outside; b and c). The empty 30–34-year age band among never internet users in 2023 accounts for 11.61% of the target and is included in the 14.43% age-range gap. Joint age-by-sex-by-education-by-hukou support diagnostics are in Supplementary Table S9.

Corresponding differences were 10.09 points in 2017 (95% CI 7.81–12.38), 8.35 in 2018 (4.85–11.85), 5.04 in 2021 (1.37–8.71), and 5.16 in 2023 (−0.05–10.38). All nine point estimates were positive, ranging from 2.12 to 10.09 points; intervals included zero in 2010 and 2023. Binary probability ratios ranged from 1.117 in 2010 to 1.428 in 2017. In 2023, the ratio was 1.193 (95% CI 1.007 to 1.413): its interval excluded 1 whereas the difference interval included 0. These nonlinear Wald approximations depend on the chosen scale near the null (Supplementary Table S6A).

The auxiliary equal-wave standardized probability difference for the core-adjusted binary comparison across the nine A30 waves was 5.99 percentage points. Applying six-point multipliers to province-level influence contributions within each wave over 99,999 draws gave a 95% CI of 4.84 to 7.16 points; the full-estimator jackknife, with refitting after each of 233 wave-by-province deletions, gave a 95% CI of 4.79 to 7.20 points (Supplementary Table S13B).

All three internet-use groups were represented in every wave. The smallest group was low frequency users in 2011, with 590 records and a Kish effective sample size of 424.7 (Figures 3b–d, Supplementary Tables S7, S9).

Frequent-minus-never internet-use differences ranged from 4.29 points in 2010 (95% CI 1.17–7.42) to 13.65 in 2017 (10.82–16.47), with positive intervals in every wave. Standardized probabilities for never, low frequency and frequent users were 16.99%, 21.48% and 21.28% in 2010 and 20.71%, 27.29% and 34.35% in 2017. The corresponding frequent-to-never probability ratios ranged from 1.238 in 2023 to 1.659 in 2017; all three contrasts are reported on both scales in Supplementary Table S7B.

Frequent-minus-low-frequency differences ranged from −0.19 points in 2010 (95% CI −2.93–2.54) to 7.07 in 2017 (4.11–10.03); the 2018 estimate was 6.61 (2.47–10.74), while the other seven intervals included zero. Low-frequency-minus-never estimates ranged from 1.54 in 2023 (−2.10–5.19) to 8.32 in 2012 (4.15–12.50).

In 2023, standardized probabilities were 25.80%, 27.34% and 31.95% for never, low frequency and frequent internet users. Frequent-minus-never and frequent-minus-low-frequency differences were 6.15 points (95% CI 0.81–11.50) and 4.61 (−0.95–10.17). All A30 respondents that year were aged 30–74, and those aged 30–35 accounted for 14.43% of the weighted population; observed never users, however, were aged 36–74. Standardizing the never-use exercise probability to all A30 respondents therefore required model extrapolation for ages 30–35, where no never users were observed (Figures 3b, c, Supplementary Table S9).

### 4.4 Exercise probabilities and differences across the five response categories

Five-category exercise probabilities did not consistently increase with internet-use frequency. Probabilities for often users (response 4) and always/very frequent users (response 5) were 34.40% and 28.48% in 2011, and 29.20% and 33.73% in 2023. The highest-frequency-minus-often contrast was −5.92 percentage points in 2011 (95% CI −10.96 to −0.89) and 4.52 in 2023 (1.45 to 7.59). Intervals for sometimes (response 3) minus rarely (response 2) users included zero in every wave (Supplementary Table S8).

The five groups also differed in the ages and social characteristics actually observed. In 2011, often users (response 4) were aged 30–69, whereas 7.72% of the full weighted A30 population were older; sometimes users (response 3) had no observations in the lowest education group (no formal schooling, traditional private schooling or literacy classes), which comprised 14.51% of the full weighted A30 population. In 2023, sometimes users (response 3) were aged 33 or older, whereas 5.87% of the full weighted A30 population were younger than 33. All five probabilities and four adjacent-category contrasts per wave were reported alongside these coverage diagnostics (Supplementary Tables S8, S9).

### 4.5 Age and social-profile gaps in the observed comparison groups

All 18 three- and five-category models converged with full-rank matrices and no fitting warnings, but successful fitting did not imply that every comparison population was represented in each group. Additional observed-support gaps included 3.13% of the weighted 2021 target below the never users' minimum age of 31, and 1.55% of the 2011 target above frequent users' maximum age of 73. In 2010, no frequent internet user was observed in Tibet, which accounted for 0.20% of the weighted target (Supplementary Table S9).

Cross-classifying 5-year age bands, sex, education and hukou further assessed whether groups included respondents with the same combination of social characteristics. In the full weighted A30 population, the share falling in combinations with no frequent-user records was 24.47% in 2010 and 34.91% in 2011. In 2023, shares in combinations with no never-, low-frequency- or frequent-user records were 37.25%, 10.08% and 0.08%, respectively. These percentages measure gaps in observed coverage within a comparison group, not missing respondent information; joint and single-characteristic gaps overlap and cannot be added.

### 4.6 Same-sample adjustment comparisons and sensitivity to unmeasured confounding

In C30, all three binary models used the same 64,000 records and within-wave targets. The auxiliary equal-wave difference was 12.66 points without adjustment (95% CI 11.14 to 14.17), 5.98 with core adjustment (4.76 to 7.20), and 5.31 after additionally adjusting for marital status, employment and household-income rank (4.09–6.54) (Supplementary Table S10).

Restriction from A30 to C30 changed the equal-wave core-adjusted estimate from 5.99 to 5.98 percentage points (−0.01 points). Adding the extended variables within C30 changed it by −0.67 points, distinguishing the changes associated with sample restriction and additional adjustment.

In the multiply imputed sensitivity analyses, the auxiliary equal-wave difference was 5.38 percentage points (95% CI 4.23 to 6.54) with extended adjustment and 5.14 (3.98 to 6.31) after additionally conditioning on self-rated health (Supplementary Table S10D).

For the core-adjusted binary comparisons in A30, thresholds were 1.480–2.209 for the point estimates and 1.000–1.891 for the confidence limits closest to the null (Supplementary Table S6B). These are necessary rather than sufficient conditions: confounding of these strengths would not necessarily explain the observed results.

## 5 Discussion

### 5.1 Interpreting changes in the composition of nonfrequent and frequent users

Across the nine surveys, understanding changes in nonfrequent internet users required considering both their internal frequency mixture and their social characteristics. The nonfrequent group changed from predominantly never use in 2010 to similar weighted shares of never and low-frequency use in 2023; frequent users also selected the highest-frequency response more often. Alongside changes in age, education, hukou and income, these findings show that the same binary comparison involved different behavioral mixtures and social populations across years. This connects diffusion theory's attention to group differences (3) and non-use research's attention to non-users' characteristics (5, 9) to the interpretation of exercise associations across years.

The changes were dimension-specific: nonfrequent users became older and occupied a lower relative income position, while their gaps from frequent users narrowed for education, hukou and income but widened for age. Frequent use also reached a broader range of educational backgrounds. Its share among tertiary-educated adults increased even as their share among frequent users declined; among junior-secondary-educated adults, frequent use rose from 11.05% to 74.92% between 2010 and 2023 (Supplementary Table S5). These results show a wider range of educational backgrounds among frequent users. Research distinguishing usage, skills, beneficial activities and material access shows why diffusion of use frequency and equality in digital skills or benefits are different questions (10, 26–28).

The endpoint ages also suggest a cohort-related question. In 2010 and 2023, nonfrequent internet users had weighted mean ages of 49.35 and 62.39 years, respectively, an increase close to the 13-year interval between surveys. The corresponding weighted mean birth years were approximately 1960.65 and 1960.61. This suggests that earlier-born cohorts may continue to account for a substantial share of nonfrequent users. Similar mean birth years do not, however, establish a stable cohort composition; that interpretation requires examining the full birth-cohort distributions.

### 5.2 Interpreting exercise associations through finer groupings and adjustment

All nine binary point estimates were positive, although their magnitudes and uncertainty varied. Frequent-versus-never differences were consistently larger than frequent-versus-low-frequency differences; the highest frequency response did not consistently have the highest exercise probability. The binary association therefore combines comparisons among behaviorally and socially different groups. Interpretation rests on the estimated differences and their uncertainty rather than changes in significance labels (29).

Adjustment comparisons within the same sample further inform the associations between internet use and exercise reported in Chinese studies (15, 30, 31). In C30, the equal-wave exercise probability difference was smaller after core adjustment and decreased further after adjustment for marital status, employment and household income. Because these comparisons used identical respondents and weights, the reductions corresponded to additional adjustment rather than a change in the analyzed population. This finding is consistent with the shared social conditions emphasized by health-lifestyle theory (12), indicating that age, education and economic characteristics are important to interpreting the group differences. The remaining adjusted association may still reflect both daily activity arrangements and unmeasured population characteristics.

The leisure domain matters. China Health and Nutrition Survey evidence links declining total activity mainly to occupational and domestic activity (32), with income and urbanicity associations varying by domain and year (33). Leisure activity also differs by age, residence and income (34). Internet use and leisure exercise can thus coexist within changes in work, resources and daily routines, even when total activity follows another pattern.

### 5.3 Interpreting cross-year comparisons and extending single-year research

The nine survey waves allow the association between internet use and leisure exercise to be interpreted in the context of population composition and survey settings in different years. The populations represented by the same use-frequency categories vary across years, and comparisons of their population shares also require consideration of weighting and survey coverage. For example, frequent internet use in D18 decreased between 2021 and 2023 in primary-weighted estimates but increased in unweighted estimates (Supplementary Table S3D), indicating that the direction of this short-term descriptive change is sensitive to weighting. Behavioral interpretations of the change between these two waves therefore require further evidence. Reporting the population composition of use categories alongside their exercise associations across multiple waves provides a broader context for interpreting findings from a single survey year.

This study extends single-year research by examining who is being compared alongside the magnitude of exercise differences. Relative to our earlier CGSS2023 article on a one-category internet-frequency contrast across several outcomes (31), it adds eight survey years and reports binary, three-category and five-category results together with population composition and observed coverage of the comparison groups. The 2023 analysis shares 4,323 of its 4,521 records with that article's 5,621-record sample, so this evidence extends the earlier study rather than constituting independent replication.

### 5.4 Limitations

The repeated cross-sections and concurrent self-reports cannot separate causal effects, health selection, individual adoption, cohort replacement or pandemic-related period influences. The media-battery internet item measures frequency, not purpose, duration, skills or benefits; leisure-exercise frequency does not capture intensity or total activity. Device examples and top-response wording varied across years, so stable coding alone does not establish measurement invariance. Grouping and adjustment comparisons are not causal decompositions. Shared response tendencies and unmeasured general lifestyle factors may still influence the observed association.

Some standardized comparisons required model extrapolation because a use group lacked observations for ages or combinations of social characteristics present in the target population. Province clustering approximates the unrecovered official sampling design; changes in geographic coverage and released weights may affect cross-year descriptions. Cross-wave respondent or household recurrence is unknown. Missingness and participation may introduce selection, and imputation assumes conditional missing at random and compatible models.

The E-value-formula thresholds for the binary comparisons are conditional on the outcome-model predictions, including extrapolation beyond observed support; they address neither measurement error nor extrapolation bias and are not exact minimum confounding thresholds for eliminating a marginal average causal effect.

Each wave uses its own standardization population; auxiliary averages describe different survey settings, not change within a fixed population. Pointwise exploratory intervals do not provide simultaneous coverage or establish equivalence. No equivalence margin was specified, and neither interval overlap nor differing significance tests equality across years (29).

## 6 Conclusion

Across nine surveys, the same nonfrequent internet-use label encompassed different frequency mixtures and social populations. Frequent internet use was positively associated with leisure exercise in all binary point estimates, with magnitudes and support varying across years and comparison groups. Separating never from low frequency users clarified these comparisons. Reporting who each frequency group represents alongside its exercise association provides a more informative basis for cross-year interpretation.

## Data Availability

The datasets are available through the China National Social Survey Data Archive (CNSSDA): http://nsrc.ruc.edu.cn/Home/Data_Resources/Chinese_National_Social_Survey_Data_Archive/.
