# Supplementary material for: Changing social composition of internet-use frequency categories and leisure exercise in China: a repeated cross-sectional study
Source: Front Public Health. 2026 Oct 22;14:1965960. doi: 10.3389/fpubh.2026.1965960 (PMC13736595; doi:10.3389/fpubh.2026.1965960)
Supplement: Supplementary file 1 [file Data_Sheet_1.docx]

Supplementary information

Changing social composition of internet-use frequency categories and leisure exercise in China: a repeated cross-sectional study

Supplementary Tables S1–S13 document questionnaire harmonization, sample construction, category composition, standardized associations, comparison support and sensitivity analyses for the nine CGSS surveys. Counts refer to respondent records. The outcome is leisure exercise daily or several times per week unless an alternative threshold is explicitly named. Probability differences are expressed in percentage points; category proportions and standardized outcome probabilities have different denominators. Probability ratios are dimensionless and divide two standardized exercise probabilities from the same wave and model.

Table notes define the samples, denominators and interval methods. Numeric citations refer to the accompanying main manuscript; references prefixed SR are listed at the end of this supplement.

## Contents

Supplementary Table S1. Measures, weights and questionnaire harmonization

Supplementary Table S2. Sample construction, item missingness and 2023 routing

Supplementary Table S3. Internet-use distributions and mixtures within binary categories

Supplementary Table S4. Social composition within internet-use categories and valid denominators

Supplementary Table S5. Internet-use categories within social groups

Supplementary Table S6. Binary standardized exercise associations and sensitivity thresholds

Supplementary Table S7. Three-category standardized exercise probabilities and contrasts

Supplementary Table S8. Five-category exercise probabilities and adjacent-category contrasts

Supplementary Table S9. Observed comparison support and model diagnostics

Supplementary Table S10. Adjustment on identical records and missing-data sensitivities

Supplementary Table S11. Equal-wave summaries and model-specification sensitivity

Supplementary Table S12. Age, geographic scope, weighting, definitions and interview mode

Supplementary Table S13. Survey-design information, finite-cluster inference and deletion checks

# Supplementary Table S1. Measures, weights and questionnaire harmonization

Harmonization began with the official questionnaire for each included wave and the official codebook where available. Public-use field names and value labels were reconciled to those documents. Waves were retained when the reference period, behavioral domain, ordered scale and threshold-defining anchors were preserved. Device examples, punctuation, field names and non-substantive response codes were documented; mode, routing and changes in the behavior measured were treated as substantive discontinuities.

The substantive A28 stem is “过去一年，您对以下媒体的使用情况是” (“In the past year, how often did you use the following media?”). Its internet item provides the frequency measure used here. Its five substantive responses are never (1), rarely (2), sometimes (3), often (4), and always/very frequently (5). The binary classification compares frequent (4–5) with nonfrequent (1–3) use; the three-category classification separates never (1), low frequency (2–3), and frequent use (4–5). The five-category model treats the responses as factor levels without assuming equal spacing.

A30 item 9 asks about physical exercise during leisure time in the past year. Its five responses are daily, several times per week, several times per month, several times per year or less, and never. Codes 1–2 define the exercise outcome: daily or several times per week; codes 3–5 define the comparison outcome. There is no separate once-per-week response, and this binary outcome does not measure guideline attainment.

## S1A. Questionnaire and official-weight locators

| **Year / mode** | **Questionnaire PDF page (printed)** | **A28 / A30 item 9 fields** | **Official weight** | **Decision** |
| --- | --- | --- | --- | --- |
| 2010, in person | p. 8 (7) | a285 / a3009 | weight | Retained: upper anchor “always” |
| 2011, in person | p. 9 (7) | a285 / a3009 | weight | Retained: mobile phone/iPad examples |
| 2012, forms A and B | p. 8 (6) in both forms | a285 / a3009 | weight | Retained: A/B focal content identical |
| 2013, forms A and B | p. 8 (6) in both forms | a285 / a3009 | weight_raking | Retained: A/B focal content identical |
| 2015, in person | p. 8 (8) | a285 / a3009 | weight_raking | Retained: added administrative code |
| 2017, in person | p. 10 (9) | a285 / a309 | weight_raking | Retained |
| 2018, in person | A28 p. 22 (20); A30 p. 23 (21) | a285 / a309 | weight_raking | Retained |
| 2021, in person | A28 p. 21 (19); A30 p. 22 (20) | A28_5 / A30_9 | weight_raking | Retained |
| 2023, in person | A28 p. 9 (7); A30 pp. 9–10 (7–8) | a285 / a3009 | weight1 | Retained: semantic labels reconcile |
| 2023, telephone | A28 p. 3 (2); A30 p. 4 (3) | a285 / a3009 | weight2 for the mixed-mode bridge | Separate mode/routing bridge only |

The two field names are listed in the order A28 internet use / A30 item 9 leisure exercise. The 2023 telephone weight is the integrated weight used for the mixed-mode bridge.

Do-not-know, refusal, not-applicable and unable responses are excluded by their semantic labels; their numeric codes changed across releases. In 2023, questionnaire values 98/99 correspond to public-use values −2/−3. Telephone routing and structural non-asking are treated separately (S2E). Changes in device examples, punctuation and field names do not alter the selected frequency threshold. The 2012/2013 forms A/B have identical focal wording, but their assignment mechanism is undocumented. A15a in 2017/2018 measures 30-minute sweat-inducing sessions and is not combined with A30. Available documentation did not establish the full construction or intended population of pre-2023 released weights; those weights are used without an undocumented interpretation. No documentary difference required exclusion of a primary in-person wave. Comparability at the selected threshold does not establish latent measurement invariance (SR1, SR2).

# Supplementary Table S2. Sample construction, item missingness and 2023 routing

D18 includes primary in-person respondents with valid ages 18–110, valid A28 and positive finite official primary weights. D30 restricts D18 to ages 30–74. A30 additionally requires a valid exercise outcome and complete core covariates. C30 comprises A30 records complete for marital status, employment and household income. G10_D18 and G10_D30 restrict the corresponding descriptive frames to the ten provinces observed in every wave: Anhui, Fujian, Gansu, Guangxi, Hebei, Henan, Jiangxi, Liaoning, Shanxi and Chongqing. Age is survey year minus birth year; the 2018 Shenzhen category is combined with Guangdong. Counts are respondent records in repeated cross-sections.

## S2A. Descriptive and association sample counts

| **Year** | **D18** | **D30** | **A30** | **C30** | **A30 Never** | **A30 Low** | **A30 Frequent** |
| --- | --- | --- | --- | --- | --- | --- | --- |
| 2010 | 11,708 | 9,376 | 9,307 | 8,327 | 6,707 | 1,190 | 1,410 |
| 2011 | 5,593 | 4,461 | 4,437 | 4,018 | 3,208 | 590 | 639 |
| 2012 | 11,753 | 9,370 | 9,345 | 8,427 | 6,226 | 1,338 | 1,781 |
| 2013 | 11,413 | 9,013 | 8,977 | 7,907 | 5,641 | 1,522 | 1,814 |
| 2015 | 10,917 | 8,478 | 8,424 | 7,798 | 4,873 | 1,367 | 2,184 |
| 2017 | 12,574 | 9,904 | 9,878 | 9,082 | 4,381 | 1,453 | 4,044 |
| 2018 | 12,780 | 10,094 | 10,066 | 9,125 | 3,872 | 1,768 | 4,426 |
| 2021 | 8,141 | 6,288 | 6,263 | 5,129 | 1,707 | 1,020 | 3,536 |
| 2023 | 5,578 | 4,528 | 4,521 | 4,187 | 768 | 870 | 2,883 |
| Total | 90,457 | 71,512 | 71,218 | 64,000 | 37,383 | 11,118 | 22,717 |

Counts are unweighted respondent records. D18: age ≥18, valid internet-use response and positive finite weight; D30: ages 30–74 within D18; A30: D30 with valid exercise outcome and complete core covariates; C30: complete extended-adjustment sample. Totals are 90,457, 71,512, 71,218 and 64,000, respectively. These are repeated cross-sections, not follow-up counts of unique people.

## S2B. Sequential construction of D18

| **Year** | **Release n** | **Mode excluded** | **Age excluded** | **Weight excluded** | **A28 excluded** | **D18 n** |
| --- | --- | --- | --- | --- | --- | --- |
| 2010 | 11,783 | 0 | 9 | 0 | 66 | 11,708 |
| 2011 | 5,620 | 0 | 1 | 0 | 26 | 5,593 |
| 2012 | 11,765 | 0 | 4 | 0 | 8 | 11,753 |
| 2013 | 11,438 | 0 | 2 | 0 | 23 | 11,413 |
| 2015 | 10,968 | 0 | 0 | 34 | 17 | 10,917 |
| 2017 | 12,582 | 0 | 0 | 0 | 8 | 12,574 |
| 2018 | 12,787 | 0 | 1 | 0 | 6 | 12,780 |
| 2021 | 8,148 | 0 | 0 | 0 | 7 | 8,141 |
| 2023 | 11,326 | 5,743 | 0 | 0 | 5 | 5,578 |

Exclusions are counted at their first sequential step: primary mode, valid age ≥18, positive finite weight, then valid A28. This order differs from the A30 flow below.

## S2C. Sequential construction of A30

| **Screening step** | **2010** | **2011** | **2012** | **2013** | **2015** | **2017** | **2018** | **2021** | **2023** |
| --- | --- | --- | --- | --- | --- | --- | --- | --- | --- |
| Release records | 11,783 | 5,620 | 11,765 | 11,438 | 10,968 | 12,582 | 12,787 | 8,148 | 11,326 |
| Exclude: nonprimary interview mode | 0 | 0 | 0 | 0 | 0 | 0 | 0 | 0 | 5,743 |
| Exclude: invalid or missing age | 9 | 1 | 4 | 2 | 0 | 0 | 1 | 0 | 0 |
| Exclude: age <30 years | 1,716 | 813 | 1,596 | 1,649 | 1,545 | 1,622 | 1,541 | 1,117 | 489 |
| Exclude: age >74 years | 621 | 321 | 788 | 754 | 910 | 1,050 | 1,146 | 738 | 562 |
| Exclude: invalid or missing A28 | 61 | 24 | 7 | 20 | 12 | 6 | 5 | 5 | 4 |
| Exclude: invalid or missing A30 | 48 | 16 | 16 | 23 | 24 | 13 | 13 | 11 | 7 |
| Exclude: nonpositive or missing primary weight | 0 | 0 | 0 | 0 | 21 | 0 | 0 | 0 | 0 |
| Exclude: invalid or missing sex | 0 | 0 | 0 | 0 | 0 | 0 | 0 | 0 | 0 |
| Exclude: invalid or missing ethnicity | 14 | 7 | 9 | 10 | 15 | 0 | 0 | 0 | 0 |
| Exclude: invalid or missing education | 7 | 1 | 0 | 3 | 17 | 13 | 15 | 14 | 0 |
| Exclude: invalid or missing hukou | 0 | 0 | 0 | 0 | 0 | 0 | 0 | 0 | 0 |
| Exclude: missing harmonized province | 0 | 0 | 0 | 0 | 0 | 0 | 0 | 0 | 0 |
| Final primary roster | 9,307 | 4,437 | 9,345 | 8,977 | 8,424 | 9,878 | 10,066 | 6,263 | 4,521 |

Read down each year column. Each record is counted only at its first exclusion. The 96,417 release records yield 71,218 A30 records. Mode restriction precedes outcome and covariate eligibility. This sequence differs from the D18 flow in S2B.

## S2D. Exclusions from D30 to A30

| **Year** | **Exercise** | **Ethnicity** | **Education** | **Any reason** |
| --- | --- | --- | --- | --- |
| 2010 | 48 | 14 | 7 | 69 |
| 2011 | 16 | 7 | 1 | 24 |
| 2012 | 16 | 9 | 0 | 25 |
| 2013 | 23 | 10 | 3 | 36 |
| 2015 | 22 | 15 | 17 | 54 |
| 2017 | 13 | 0 | 13 | 26 |
| 2018 | 13 | 0 | 15 | 28 |
| 2021 | 11 | 0 | 14 | 25 |
| 2023 | 7 | 0 | 0 | 7 |
| Total | 169 | 55 | 70 | 294 |

No records are excluded for sex, hukou or province at this stage. The 294 excluded records comprise 169 invalid exercise outcomes, 55 missing ethnicity values and 70 missing education values. The 2015 D30 flow excludes nonpositive weights before outcome eligibility, giving 22 exercise exclusions rather than the 24 in the differently ordered A30 release flow.

## S2E. 2023 interview mode and routing

| **Indicator** | **In person** | **Telephone** |
| --- | --- | --- |
| Release records | 5,583 | 5,743 |
| A28 release-observed | 5,583 | 1,399 |
| A30 release-observed | 5,583 | 1,433 |
| A28 structural or system missing | 0 | 4,344 |
| A30 structural or system missing | 0 | 4,310 |
| Valid A28 | 5,578 | 1,398 |
| Valid A30 | 5,573 | 1,430 |
| Valid pair | 5,569 | 356 |
| Primary-roster eligible | 4,521 | 0 |
| Mixed-mode bridge eligible | 4,521 | 261 |

Release-observed means availability in the public-use field, not interviewer paradata. Structural or system missingness is distinct from an observed but invalid response.

Release-observed means availability in the public-use field, not interviewer paradata. The telephone questionnaire randomized batteries and A30 subitems, included bound-routing and termination values, and could suppress A30 after an all-never A28 battery. Structural non-asking was not imputed. The 261 complete telephone records enter only the mixed-mode bridge with the integrated weight.

# Supplementary Table S3. Internet-use distributions and mixtures within binary categories

Sample frames are defined in S2. The five-response percentages in S3A and the three-category percentages in S3B use the full D18 frame with valid A28. Internal-mixture percentages instead condition on the corresponding binary category: nonfrequent (NF) = responses 1–3 and frequent (F) = responses 4–5. Counts are unweighted; percentages use official weights unless a comparison scheme is explicitly named in S3D. Kish effective sample size (ESS) = (Σw)²/Σw² describes weight concentration and does not incorporate clustering. S3A retains response-specific ESS, while S3C provides the age-restricted and common-province comparisons.

## S3A. D18 five-response distributions and effective sample sizes

| **Year** | **Frame n** | **1 Never** | **2 Rarely** | **3 Sometimes** | **4 Often** | **5 Always/very frequently** |
| --- | --- | --- | --- | --- | --- | --- |
| 2010 | 11,708 | 7,669 (66.96) ESS 6367.05 | 788 (6.78) ESS 681.70 | 844 (7.11) ESS 733.38 | 1,261 (10.37) ESS 1098.46 | 1,146 (8.78) ESS 977.42 |
| 2011 | 5,593 | 3,640 (66.31) ESS 2578.62 | 381 (6.12) ESS 264.80 | 395 (6.36) ESS 283.27 | 572 (9.67) ESS 399.19 | 605 (11.54) ESS 423.51 |
| 2012 | 11,753 | 7,152 (62.59) ESS 5027.86 | 745 (5.63) ESS 552.67 | 888 (6.71) ESS 646.54 | 1,313 (10.50) ESS 944.06 | 1,655 (14.57) ESS 1160.92 |
| 2013 | 11,413 | 6,481 (53.00) ESS 4227.68 | 893 (7.53) ESS 568.26 | 931 (8.37) ESS 572.21 | 1,479 (14.19) ESS 910.40 | 1,629 (16.91) ESS 985.19 |
| 2015 | 10,917 | 5,814 (46.52) ESS 4146.76 | 781 (7.21) ESS 576.06 | 807 (7.47) ESS 573.60 | 1,520 (15.55) ESS 1023.55 | 1,995 (23.25) ESS 1239.75 |
| 2017 | 12,574 | 5,339 (20.55) ESS 1934.71 | 696 (4.88) ESS 235.06 | 917 (8.46) ESS 317.29 | 2,275 (25.25) ESS 840.09 | 3,347 (40.85) ESS 1311.61 |
| 2018 | 12,780 | 4,876 (16.68) ESS 1441.79 | 868 (5.91) ESS 278.87 | 1,087 (8.87) ESS 356.54 | 2,647 (29.09) ESS 970.15 | 3,302 (39.45) ESS 1252.78 |
| 2021 | 8,141 | 2,295 (11.35) ESS 827.74 | 506 (5.06) ESS 202.94 | 618 (8.11) ESS 279.50 | 1,654 (25.37) ESS 601.74 | 3,068 (50.11) ESS 1083.98 |
| 2023 | 5,578 | 1,072 (13.82) ESS 772.29 | 469 (6.49) ESS 326.65 | 532 (7.96) ESS 366.32 | 1,287 (25.82) ESS 888.32 | 2,218 (45.91) ESS 1570.25 |

Each response cell gives unweighted n (official-weighted % of D18), followed by its category-specific Kish ESS. The five percentages sum to 100% apart from rounding.

## S3B. D18 three categories and binary-category mixtures

| **Year** | **Never %** | **Low %** | **Frequent %** | **1 within NF %** | **2 within NF %** | **3 within NF %** | **5 within F %** |
| --- | --- | --- | --- | --- | --- | --- | --- |
| 2010 | 66.96 | 13.89 | 19.15 | 82.82 | 8.38 | 8.80 | 45.86 |
| 2011 | 66.31 | 12.48 | 21.21 | 84.16 | 7.77 | 8.07 | 54.42 |
| 2012 | 62.59 | 12.34 | 25.08 | 83.53 | 7.51 | 8.96 | 58.12 |
| 2013 | 53.00 | 15.90 | 31.10 | 76.93 | 10.93 | 12.14 | 54.38 |
| 2015 | 46.52 | 14.68 | 38.80 | 76.01 | 11.78 | 12.21 | 59.91 |
| 2017 | 20.55 | 13.34 | 66.11 | 60.63 | 14.41 | 24.96 | 61.80 |
| 2018 | 16.68 | 14.78 | 68.54 | 53.02 | 18.79 | 28.19 | 57.56 |
| 2021 | 11.35 | 13.17 | 75.47 | 46.30 | 20.63 | 33.07 | 66.39 |
| 2023 | 13.82 | 14.45 | 71.73 | 48.88 | 22.97 | 28.15 | 64.01 |

NF = nonfrequent, F = frequent. The three category shares use the frame denominator. Within-group percentages use only NF or F; response 4 within F is 100 minus response 5 within F.

## S3C. Age-restricted and common-province frequency comparisons

| **Year** | **Frame** | **Frame n / ESS*** | **Frequent %** | **Never within NF %** |
| --- | --- | --- | --- | --- |
| 2010 | D30 | 9,376 | 13.66 | 85.92 |
| 2010 | G10_D18 | 3,632 / 3073.48 | 14.36 | 84.15 |
| 2010 | G10_D30 | 2,955 / 2513.85 | 9.88 | 87.47 |
| 2011 | D30 | 4,461 | 14.41 | 85.59 |
| 2011 | G10_D18 | 2,246 / 1525.30 | 17.41 | 84.95 |
| 2011 | G10_D30 | 1,787 / 1245.95 | 10.46 | 86.79 |
| 2012 | D30 | 9,370 | 18.78 | 83.94 |
| 2012 | G10_D18 | 3,707 / 2585.01 | 19.39 | 83.93 |
| 2012 | G10_D30 | 2,975 / 2098.71 | 13.36 | 85.22 |
| 2013 | D30 | 9,013 | 19.08 | 79.30 |
| 2013 | G10_D18 | 3,702 / 2260.07 | 25.58 | 78.01 |
| 2013 | G10_D30 | 2,950 / 1921.32 | 14.68 | 81.37 |
| 2015 | D30 | 8,478 | 27.42 | 76.63 |
| 2015 | G10_D18 | 3,555 / 2326.85 | 37.61 | 79.07 |
| 2015 | G10_D30 | 2,764 / 1960.89 | 23.99 | 81.11 |
| 2017 | D30 | 9,904 | 50.59 | 63.78 |
| 2017 | G10_D18 | 3,721 / 1131.65 | 64.98 | 61.26 |
| 2017 | G10_D30 | 2,942 / 1482.31 | 47.60 | 63.34 |
| 2018 | D30 | 10,094 | 56.12 | 55.65 |
| 2018 | G10_D18 | 3,759 / 1087.70 | 63.56 | 50.91 |
| 2018 | G10_D30 | 3,003 / 1238.88 | 50.22 | 55.29 |
| 2021 | D30 | 6,288 | 69.51 | 42.93 |
| 2021 | G10_D18 | 4,156 / 1295.48 | 74.97 | 43.34 |
| 2021 | G10_D30 | 3,147 / 1331.21 | 68.04 | 40.83 |
| 2023 | D30 | 4,528 | 70.95 | 44.00 |
| 2023 | G10_D18 | 3,559 / 2615.59 | 73.12 | 49.25 |
| 2023 | G10_D30 | 2,892 / 2142.25 | 72.63 | 43.78 |

Frequent % uses the full named frame; Never within NF % uses only responses 1–3 in that frame. *For the two G10 frames, n and frame-level Kish ESS are separated by a slash; D30 displays n only. All nine waves are retained. G10 uses the ten provinces listed in S2.

## S3D. Descriptive weight comparisons on identical records

| **Frame / year** | **Measure** | **Primary %** | **Unweighted % Δ pp [95% CI]** | **Capped % Δ pp [95% CI]** | **Base % Δ pp [95% CI]** |
| --- | --- | --- | --- | --- | --- |
| D18 2010 | Frequent | 19.15 | 20.56 1.41 [0.46, 2.36] | 19.17 0.02 [−0.01, 0.05] | — |
| D18 2010 | NF never | 82.82 | 82.45 −0.36 [−0.90, 0.17] | 82.79 −0.03 [−0.05, −0.01] | — |
| D18 2011 | Frequent | 21.21 | 21.04 −0.16 [−1.48, 1.16] | 21.19 −0.02 [−0.05, 0.01] | — |
| D18 2011 | NF never | 84.16 | 82.43 −1.73 [−2.94, −0.52] | 84.16 0.00 [−0.04, 0.04] | — |
| D18 2012 | Frequent | 25.08 | 25.25 0.18 [−1.07, 1.42] | 25.08 0.00 [−0.01, 0.01] | — |
| D18 2012 | NF never | 83.53 | 81.41 −2.12 [−2.94, −1.30] | 83.52 −0.01 [−0.02, 0.00] | — |
| D18 2013 | Frequent | 31.10 | 27.23 −3.87 [−4.94, −2.80] | 30.77 −0.33 [−0.49, −0.17] | 26.37 −4.73 [−5.58, −3.88] |
| D18 2013 | NF never | 76.93 | 78.04 1.11 [0.19, 2.03] | 77.14 0.21 [0.08, 0.35] | 80.63 3.70 [2.87, 4.53] |
| D18 2015 | Frequent | 38.80 | 32.20 −6.60 [−7.62, −5.59] | 38.33 −0.47 [−0.64, −0.30] | 30.30 −8.50 [−9.47, −7.54] |
| D18 2015 | NF never | 76.01 | 78.55 2.53 [1.65, 3.41] | 76.10 0.09 [−0.02, 0.19] | 81.37 5.35 [4.41, 6.29] |
| D18 2017 | Frequent | 66.11 | 44.71 −21.40 [−24.60, −18.19] | 65.48 −0.63 [−1.05, −0.21] | 59.49 −6.62 [−8.72, −4.52] |
| D18 2017 | NF never | 60.63 | 76.80 16.16 [13.66, 18.66] | 61.38 0.75 [0.09, 1.40] | 69.67 9.04 [7.58, 10.49] |
| D18 2018 | Frequent | 68.54 | 46.55 −21.99 [−24.94, −19.04] | 68.14 −0.40 [−0.78, −0.01] | 61.06 −7.48 [−9.68, −5.28] |
| D18 2018 | NF never | 53.02 | 71.38 18.36 [15.05, 21.67] | 53.38 0.35 [−0.32, 1.03] | 63.81 10.79 [8.60, 12.98] |
| D18 2021 | Frequent | 75.47 | 58.00 −17.47 [−20.87, −14.07] | 74.54 −0.93 [−1.17, −0.69] | 66.66 −8.82 [−10.37, −7.27] |
| D18 2021 | NF never | 46.30 | 67.12 20.83 [18.60, 23.06] | 46.35 0.06 [−0.12, 0.23] | 58.33 12.03 [10.69, 13.38] |
| D18 2023 | Frequent | 71.73 | 62.84 −8.89 [−12.00, −5.79] | 71.73 0.00 [0.00, 0.00] | — |
| D18 2023 | NF never | 48.88 | 51.71 2.83 [−0.04, 5.71] | 48.88 0.00 [0.00, 0.00] | — |
| D30 2010 | Frequent | 13.66 | 15.13 1.47 [0.65, 2.29] | 13.68 0.02 [−0.01, 0.05] | — |
| D30 2010 | NF never | 85.92 | 84.87 −1.06 [−1.63, −0.48] | 85.90 −0.02 [−0.04, 0.00] | — |
| D30 2011 | Frequent | 14.41 | 14.39 −0.02 [−1.06, 1.03] | 14.40 −0.01 [−0.03, 0.02] | — |
| D30 2011 | NF never | 85.59 | 84.45 −1.14 [−2.34, 0.06] | 85.59 0.00 [−0.03, 0.03] | — |
| D30 2012 | Frequent | 18.78 | 19.08 0.30 [−0.59, 1.20] | 18.78 0.00 [−0.01, 0.01] | — |
| D30 2012 | NF never | 83.94 | 82.31 −1.62 [−2.45, −0.79] | 83.92 −0.01 [−0.02, 0.00] | — |
| D30 2013 | Frequent | 19.08 | 20.22 1.13 [0.17, 2.10] | 19.07 −0.01 [−0.06, 0.03] | 19.43 0.35 [−0.50, 1.19] |
| D30 2013 | NF never | 79.30 | 78.71 −0.60 [−1.44, 0.25] | 79.30 0.00 [−0.04, 0.04] | 80.81 1.51 [0.89, 2.12] |
| D30 2015 | Frequent | 27.42 | 25.86 −1.56 [−2.18, −0.95] | 27.33 −0.09 [−0.15, −0.04] | 24.44 −2.98 [−3.77, −2.19] |
| D30 2015 | NF never | 76.63 | 78.09 1.46 [0.71, 2.22] | 76.67 0.04 [−0.01, 0.09] | 80.17 3.54 [2.71, 4.37] |
| D30 2017 | Frequent | 50.59 | 40.92 −9.67 [−12.57, −6.76] | 50.58 −0.01 [−0.02, 0.00] | 49.77 −0.82 [−3.20, 1.56] |
| D30 2017 | NF never | 63.78 | 75.12 11.34 [8.94, 13.74] | 63.78 0.00 [0.00, 0.01] | 69.93 6.15 [4.94, 7.36] |
| D30 2018 | Frequent | 56.12 | 43.98 −12.14 [−14.68, −9.61] | 56.09 −0.03 [−0.09, 0.04] | 53.87 −2.25 [−4.59, 0.09] |
| D30 2018 | NF never | 55.65 | 68.59 12.95 [10.16, 15.74] | 55.72 0.07 [−0.04, 0.19] | 63.29 7.64 [5.77, 9.52] |
| D30 2021 | Frequent | 69.51 | 56.44 −13.07 [−16.77, −9.37] | 69.51 0.00 [0.00, 0.00] | 64.13 −5.38 [−7.14, −3.62] |
| D30 2021 | NF never | 42.93 | 62.65 19.72 [17.66, 21.78] | 42.93 0.00 [0.00, 0.00] | 53.58 10.64 [9.40, 11.89] |
| D30 2023 | Frequent | 70.95 | 63.76 −7.19 [−10.72, −3.66] | 70.95 0.00 [0.00, 0.00] | — |
| D30 2023 | NF never | 44.00 | 46.86 2.86 [0.15, 5.57] | 44.00 0.00 [0.00, 0.00] | — |

Frequent uses all valid A28 responses in the named frame; NF never is response 1 among responses 1–3. In each alternative cell, the first line is the percentage and the following line is alternative minus primary in percentage points with its paired 95% CI. The same records are used throughout each wave/frame; N is given in S2. Primary weights are weight (2010–2012), weight_raking (2013, 2015, 2017, 2018 and 2021), and weight1 (2023 in person). Base denotes the released weight field in the five raking waves; field labels do not establish identical weight construction across releases. —: no comparable base branch specified; 2023 weight2 is an integrated weight and is not used as base. Capping replaces primary weights below/above the D18 wave-specific 1st/99th percentiles (R type 7) with those thresholds; D30 uses the same thresholds. Intervals condition on the observed weights and thresholds. Paired linearized contributions retain covariance between schemes and use province as a geographic-cluster proxy with t_(G−1) intervals. D18/D30 province counts G, in year order, are 31, 26, 29, 28, 28, 28, 28, 19 and 16 in both frames. These are within-wave weight comparisons, not tests of between-wave change. Zero displayed changes may reflect rounding; in 2023 capping changes no weights. Different weighting rules define different descriptive targets and do not identify a preferred true population estimate.

# Supplementary Table S4. Social composition within internet-use categories and valid denominators

Composition is conditional on the internet-use category, in contrast to the social-group denominator in Supplementary Table S5. Each variable uses its own valid-response denominator without requiring all composition variables to be complete. Age is a weighted mean; education and hukou are weighted percentages. The overall reference is the same-wave population in the named frame with the relevant variable observed. These are distributional summaries; baseline significance tests are not used.

Personal annual income is retained when nonnegative and below 9,999,990. Its weighted midpoint cumulative rank is computed among valid-income D18 respondents in each wave: the total weight below an income value plus half the weight tied at that value, divided by total valid-income weight. The same ranks are retained in D30 rather than reranked within categories or the age-restricted subset. Tables display 100 times the 0–1 rank. The D18 overall mean is 50 by construction; ties mean that rank quartiles need not contain exactly 25% of weighted records.

## S4A. D18 binary-category social profiles and overall reference

| **Year** | **Age (years)** | **Tertiary education (%)** | **Agricultural hukou (%)** | **Income percentile** |
| --- | --- | --- | --- | --- |
| 2010 | 49.35 / 33.89 / 46.39 | 5.11 / 49.59 / 13.63 | 65.22 / 24.30 / 57.39 | 45.75 / 69.68 / 50.00 |
| 2011 | 54.45 / 33.95 / 50.10 | 5.04 / 49.54 / 14.48 | 60.14 / 31.07 / 53.98 | 45.49 / 68.04 / 50.00 |
| 2012 | 55.94 / 35.15 / 50.73 | 5.32 / 49.15 / 16.31 | 59.89 / 29.04 / 52.15 | 43.87 / 68.92 / 50.00 |
| 2013 | 53.13 / 31.55 / 46.42 | 4.41 / 42.03 / 16.10 | 62.33 / 42.71 / 56.23 | 43.95 / 64.08 / 50.00 |
| 2015 | 54.81 / 33.31 / 46.47 | 3.36 / 35.82 / 15.96 | 65.01 / 46.10 / 57.68 | 43.25 / 60.76 / 50.00 |
| 2017 | 48.29 / 31.29 / 37.05 | 2.76 / 30.15 / 20.86 | 76.53 / 60.16 / 65.71 | 41.66 / 54.36 / 50.00 |
| 2018 | 49.04 / 32.21 / 37.51 | 3.37 / 29.48 / 21.26 | 78.15 / 64.01 / 68.45 | 39.29 / 54.99 / 50.00 |
| 2021 | 53.30 / 36.17 / 40.37 | 2.24 / 16.62 / 13.10 | 71.16 / 63.15 / 65.12 | 38.32 / 53.78 / 50.00 |
| 2023 | 62.39 / 43.31 / 48.71 | 3.93 / 29.85 / 22.52 | 74.80 / 62.35 / 65.87 | 37.62 / 54.89 / 50.00 |

Every cell lists Nonfrequent / Frequent / Overall, in that order. Age and income are weighted means; education and hukou are weighted percentages. Each variable uses its valid-response denominator. The overall reference is the same-wave D18 population. A valid exercise outcome is not required.

## S4B. D18 three-category profiles and overall reference

| **Year** | **Category** | **Age mean** | **Tertiary %** | **Agricultural %** | **Income percentile** | **Income valid n** | **Income missing n** |
| --- | --- | --- | --- | --- | --- | --- | --- |
| 2010 | Never | 51.51 | 2.31 | 70.21 | 42.88 | 6,800 | 869 |
| 2010 | Low | 38.91 | 18.58 | 41.18 | 60.16 | 1,381 | 251 |
| 2010 | Frequent | 33.89 | 49.59 | 24.30 | 69.68 | 1,921 | 486 |
| 2010 | Overall | 46.39 | 13.63 | 57.39 | 50.00 | 10,102 | 1,606 |
| 2011 | Never | 56.86 | 2.90 | 63.97 | 42.53 | 3,326 | 314 |
| 2011 | Low | 41.64 | 16.43 | 39.80 | 61.70 | 689 | 87 |
| 2011 | Frequent | 33.95 | 49.54 | 31.07 | 68.04 | 1,003 | 174 |
| 2011 | Overall | 50.10 | 14.48 | 53.98 | 50.00 | 5,018 | 575 |
| 2012 | Never | 58.35 | 2.41 | 64.07 | 40.47 | 6,541 | 611 |
| 2012 | Low | 43.71 | 20.05 | 38.68 | 61.28 | 1,462 | 171 |
| 2012 | Frequent | 35.15 | 49.15 | 29.04 | 68.92 | 2,617 | 351 |
| 2012 | Overall | 50.73 | 16.31 | 52.15 | 50.00 | 10,620 | 1,133 |
| 2013 | Never | 56.78 | 2.19 | 65.00 | 40.39 | 5,907 | 574 |
| 2013 | Low | 40.98 | 11.81 | 53.42 | 56.28 | 1,615 | 209 |
| 2013 | Frequent | 31.55 | 42.03 | 42.71 | 64.08 | 2,696 | 412 |
| 2013 | Overall | 46.42 | 16.10 | 56.23 | 50.00 | 10,218 | 1,195 |
| 2015 | Never | 58.15 | 1.62 | 67.79 | 39.83 | 5,532 | 282 |
| 2015 | Low | 44.23 | 8.88 | 56.20 | 54.20 | 1,497 | 91 |
| 2015 | Frequent | 33.31 | 35.82 | 46.10 | 60.76 | 3,288 | 227 |
| 2015 | Overall | 46.47 | 15.96 | 57.68 | 50.00 | 10,317 | 600 |
| 2017 | Never | 53.64 | 1.15 | 78.57 | 38.10 | 5,074 | 265 |
| 2017 | Low | 40.05 | 5.25 | 73.39 | 47.20 | 1,529 | 84 |
| 2017 | Frequent | 31.29 | 30.15 | 60.16 | 54.36 | 5,288 | 334 |
| 2017 | Overall | 37.05 | 20.86 | 65.71 | 50.00 | 11,891 | 683 |
| 2018 | Never | 55.83 | 1.18 | 80.80 | 34.85 | 4,644 | 232 |
| 2018 | Low | 41.37 | 5.83 | 75.15 | 44.37 | 1,868 | 87 |
| 2018 | Frequent | 32.21 | 29.48 | 64.01 | 54.99 | 5,588 | 361 |
| 2018 | Overall | 37.51 | 21.26 | 68.45 | 50.00 | 12,100 | 680 |
| 2021 | Never | 61.40 | 0.69 | 70.59 | 35.77 | 2,078 | 217 |
| 2021 | Low | 46.31 | 3.59 | 71.65 | 40.52 | 1,018 | 106 |
| 2021 | Frequent | 36.17 | 16.62 | 63.15 | 53.78 | 4,230 | 492 |
| 2021 | Overall | 40.37 | 13.10 | 65.12 | 50.00 | 7,326 | 815 |
| 2023 | Never | 67.72 | 1.67 | 78.38 | 33.46 | 1,027 | 45 |
| 2023 | Low | 57.30 | 6.08 | 71.38 | 41.61 | 958 | 43 |
| 2023 | Frequent | 43.31 | 29.85 | 62.35 | 54.89 | 3,370 | 135 |
| 2023 | Overall | 48.71 | 22.52 | 65.87 | 50.00 | 5,355 | 223 |

All nine waves and all three internet-use categories are shown. Each indicator uses its own valid-response denominator; income valid and missing counts are shown by category. Income uses the common wave-specific D18 personal-income rank. Overall valid and missing counts for each indicator are in S4C.

## S4C. D18 variable-specific valid and missing counts

| **Year** | **Total n** | **Age valid / missing** | **Education valid / missing** | **Hukou valid / missing** | **Income valid / missing** |
| --- | --- | --- | --- | --- | --- |
| 2010 | 11,708 | 11,708 / 0 | 11,696 / 12 | 11,705 / 3 | 10,102 / 1,606 |
| 2011 | 5,593 | 5,593 / 0 | 5,591 / 2 | 5,593 / 0 | 5,018 / 575 |
| 2012 | 11,753 | 11,753 / 0 | 11,749 / 4 | 11,753 / 0 | 10,620 / 1,133 |
| 2013 | 11,413 | 11,413 / 0 | 11,407 / 6 | 11,413 / 0 | 10,218 / 1,195 |
| 2015 | 10,917 | 10,917 / 0 | 10,891 / 26 | 10,917 / 0 | 10,317 / 600 |
| 2017 | 12,574 | 12,574 / 0 | 12,553 / 21 | 12,574 / 0 | 11,891 / 683 |
| 2018 | 12,780 | 12,780 / 0 | 12,751 / 29 | 12,780 / 0 | 12,100 / 680 |
| 2021 | 8,141 | 8,141 / 0 | 8,120 / 21 | 8,141 / 0 | 7,326 / 815 |
| 2023 | 5,578 | 5,578 / 0 | 5,577 / 1 | 5,578 / 0 | 5,355 / 223 |

Counts are unweighted records in the same-wave D18 frame. Missingness is handled separately for each composition variable. Missing income does not exclude a record from D18 or D30.

## S4D. D30 endpoint social profiles

| **Year** | **Category** | **Age mean** | **Tertiary %** | **Agricultural %** | **Income percentile** | **Income valid n** | **Income missing n** |
| --- | --- | --- | --- | --- | --- | --- | --- |
| 2010 | Never | 51.11 | 2.18 | 70.47 | 43.10 | 6,019 | 734 |
| 2010 | Low | 44.64 | 20.08 | 31.70 | 64.76 | 1,047 | 157 |
| 2010 | Frequent | 41.16 | 49.04 | 15.08 | 76.32 | 1,201 | 218 |
| 2010 | Overall | 48.96 | 10.76 | 58.19 | 50.08 | 8,267 | 1,109 |
| 2023 | Never | 63.65 | 0.33 | 81.69 | 33.16 | 730 | 39 |
| 2023 | Low | 56.66 | 4.83 | 72.89 | 41.41 | 836 | 36 |
| 2023 | Frequent | 47.50 | 21.70 | 62.47 | 57.93 | 2,788 | 99 |
| 2023 | Overall | 51.06 | 16.23 | 66.62 | 52.10 | 4,354 | 174 |

The 2010 and 2023 endpoints provide the age-restricted comparison for all four social indicators and all three categories, with the same-wave overall reference. Each indicator uses its valid-response denominator. Income retains the D18 reference and is displayed on the 0–100 scale; it is not reranked within D30. For the D30 nonfrequent category (responses 1–3), weighted mean age was 52.3 years in 2021 and 59.7 years in 2023. These are descriptive D30 estimates, before requiring a valid exercise outcome.

# Supplementary Table S5. Internet-use categories within social groups

Each row conditions on a social group in the named wave of D18. Its denominator comprises records with that social-group value and valid A28. These tables describe internet-use categories within age, education, hukou and income groups, whereas S4 describes social composition within internet-use categories. All nine waves and every category of the four social variables are retained. Missing social-group values are excluded only from that variable.

## S5A. D18: Age and hukou groups

| **Year** | **Social group** | **Valid-group n** | **Never %** | **Low %** | **Frequent %** |
| --- | --- | --- | --- | --- | --- |
| 2010 | Age: 18-29 | 1,712 | 21.24 | 26.11 | 52.65 |
| 2010 | Age: 30-44 | 3,593 | 58.96 | 16.95 | 24.09 |
| 2010 | Age: 45-59 | 3,693 | 80.72 | 10.35 | 8.92 |
| 2010 | Age: 60-74 | 2,090 | 90.89 | 6.38 | 2.73 |
| 2010 | Age: 75+ | 620 | 95.85 | 2.47 | 1.68 |
| 2010 | Hukou: Agricultural | 6,028 | 81.93 | 9.97 | 8.10 |
| 2010 | Hukou: Other | 5,677 | 46.82 | 19.19 | 33.99 |
| 2011 | Age: 18-29 | 813 | 10.49 | 19.06 | 70.44 |
| 2011 | Age: 30-44 | 1,605 | 50.02 | 19.13 | 30.85 |
| 2011 | Age: 45-59 | 1,762 | 76.41 | 13.26 | 10.33 |
| 2011 | Age: 60-74 | 1,094 | 93.24 | 3.73 | 3.02 |
| 2011 | Age: 75+ | 319 | 97.04 | 2.15 | 0.81 |
| 2011 | Hukou: Agricultural | 3,154 | 78.59 | 9.20 | 12.21 |
| 2011 | Hukou: Other | 2,439 | 51.91 | 16.33 | 31.76 |
| 2012 | Age: 18-29 | 1,596 | 7.64 | 14.62 | 77.74 |
| 2012 | Age: 30-44 | 3,311 | 40.90 | 20.26 | 38.83 |
| 2012 | Age: 45-59 | 3,598 | 72.66 | 13.33 | 14.01 |
| 2012 | Age: 60-74 | 2,461 | 89.80 | 5.36 | 4.84 |
| 2012 | Age: 75+ | 787 | 95.94 | 2.89 | 1.17 |
| 2012 | Hukou: Agricultural | 6,257 | 76.88 | 9.15 | 13.97 |
| 2012 | Hukou: Other | 5,496 | 47.00 | 15.81 | 37.19 |
| 2013 | Age: 18-29 | 1,647 | 5.56 | 16.70 | 77.74 |
| 2013 | Age: 30-44 | 3,183 | 35.88 | 25.53 | 38.59 |
| 2013 | Age: 45-59 | 3,418 | 72.58 | 15.39 | 12.03 |
| 2013 | Age: 60-74 | 2,412 | 88.55 | 7.24 | 4.21 |
| 2013 | Age: 75+ | 753 | 93.57 | 4.46 | 1.97 |
| 2013 | Hukou: Agricultural | 6,316 | 61.28 | 15.10 | 23.62 |
| 2013 | Hukou: Other | 5,097 | 42.38 | 16.92 | 40.71 |
| 2015 | Age: 18-29 | 1,534 | 3.51 | 10.55 | 85.94 |
| 2015 | Age: 30-44 | 2,481 | 25.95 | 22.05 | 52.00 |
| 2015 | Age: 45-59 | 3,330 | 63.40 | 17.20 | 19.41 |
| 2015 | Age: 60-74 | 2,667 | 82.50 | 9.87 | 7.63 |
| 2015 | Age: 75+ | 905 | 93.01 | 4.25 | 2.73 |
| 2015 | Hukou: Agricultural | 6,164 | 54.68 | 14.30 | 31.01 |
| 2015 | Hukou: Other | 4,753 | 35.40 | 15.19 | 49.41 |
| 2017 | Age: 18-29 | 1,621 | 3.14 | 7.50 | 89.36 |
| 2017 | Age: 30-44 | 2,830 | 11.45 | 18.95 | 69.59 |
| 2017 | Age: 45-59 | 3,754 | 47.28 | 19.04 | 33.69 |
| 2017 | Age: 60-74 | 3,320 | 74.38 | 9.68 | 15.95 |
| 2017 | Age: 75+ | 1,049 | 87.49 | 6.73 | 5.78 |
| 2017 | Hukou: Agricultural | 6,765 | 24.58 | 14.90 | 60.52 |
| 2017 | Hukou: Other | 5,809 | 12.84 | 10.35 | 76.81 |
| 2018 | Age: 18-29 | 1,540 | 1.85 | 8.00 | 90.15 |
| 2018 | Age: 30-44 | 2,801 | 6.92 | 17.37 | 75.71 |
| 2018 | Age: 45-59 | 3,755 | 36.92 | 24.67 | 38.41 |
| 2018 | Age: 60-74 | 3,538 | 67.45 | 14.06 | 18.48 |
| 2018 | Age: 75+ | 1,146 | 85.34 | 7.56 | 7.10 |
| 2018 | Hukou: Agricultural | 6,990 | 19.69 | 16.23 | 64.09 |
| 2018 | Hukou: Other | 5,790 | 10.15 | 11.64 | 78.21 |
| 2021 | Age: 18-29 | 1,116 | 1.18 | 3.85 | 94.97 |
| 2021 | Age: 30-44 | 1,695 | 2.19 | 13.27 | 84.54 |
| 2021 | Age: 45-59 | 2,403 | 14.54 | 23.07 | 62.39 |
| 2021 | Age: 60-74 | 2,190 | 50.98 | 17.04 | 31.97 |
| 2021 | Age: 75+ | 737 | 79.08 | 8.61 | 12.31 |
| 2021 | Hukou: Agricultural | 4,835 | 12.31 | 14.49 | 73.20 |
| 2021 | Hukou: Other | 3,306 | 9.57 | 10.71 | 79.72 |
| 2023 | Age: 18-29 | 488 | 0.44 | 3.39 | 96.17 |
| 2023 | Age: 30-44 | 1,005 | 1.33 | 5.67 | 92.99 |
| 2023 | Age: 45-59 | 1,734 | 8.64 | 20.60 | 70.76 |
| 2023 | Age: 60-74 | 1,789 | 33.04 | 23.05 | 43.91 |
| 2023 | Age: 75+ | 562 | 58.04 | 18.13 | 23.83 |
| 2023 | Hukou: Agricultural | 3,371 | 16.44 | 15.66 | 67.89 |
| 2023 | Hukou: Other | 2,207 | 8.75 | 12.12 | 79.13 |

Counts are unweighted valid-group records; percentages are official-weighted and sum to 100% within each row apart from rounding.

## S5B. D18: Education and income groups

| **Year** | **Social group** | **Valid-group n** | **Never %** | **Low %** | **Frequent %** |
| --- | --- | --- | --- | --- | --- |
| 2010 | Education: No formal schooling/private school/literacy class | 1,618 | 97.75 | 1.37 | 0.89 |
| 2010 | Education: Primary | 2,583 | 93.13 | 4.96 | 1.90 |
| 2010 | Education: Junior secondary | 3,439 | 71.26 | 17.69 | 11.05 |
| 2010 | Education: Upper secondary / vocational | 2,252 | 43.87 | 24.87 | 31.26 |
| 2010 | Education: Tertiary | 1,804 | 11.36 | 18.94 | 69.70 |
| 2010 | Income: Q1 | 2,357 | 82.52 | 8.29 | 9.19 |
| 2010 | Income: Q2 | 2,354 | 86.26 | 9.04 | 4.71 |
| 2010 | Income: Q3 | 2,970 | 64.67 | 16.71 | 18.62 |
| 2010 | Income: Q4 | 2,421 | 38.63 | 20.57 | 40.81 |
| 2011 | Education: No formal schooling/private school/literacy class | 788 | 98.24 | 1.15 | 0.61 |
| 2011 | Education: Primary | 1,297 | 92.03 | 5.08 | 2.89 |
| 2011 | Education: Junior secondary | 1,732 | 69.49 | 16.06 | 14.45 |
| 2011 | Education: Upper secondary / vocational | 984 | 43.68 | 24.48 | 31.84 |
| 2011 | Education: Tertiary | 790 | 13.28 | 14.16 | 72.56 |
| 2011 | Income: Q1 | 1,363 | 84.71 | 6.28 | 9.02 |
| 2011 | Income: Q2 | 1,350 | 81.17 | 9.28 | 9.55 |
| 2011 | Income: Q3 | 1,116 | 62.29 | 15.82 | 21.89 |
| 2011 | Income: Q4 | 1,189 | 40.50 | 18.81 | 40.69 |
| 2012 | Education: No formal schooling/private school/literacy class | 1,650 | 97.89 | 1.20 | 0.90 |
| 2012 | Education: Primary | 2,687 | 90.96 | 5.60 | 3.44 |
| 2012 | Education: Junior secondary | 3,310 | 66.33 | 16.76 | 16.92 |
| 2012 | Education: Upper secondary / vocational | 2,225 | 39.19 | 21.45 | 39.36 |
| 2012 | Education: Tertiary | 1,877 | 9.24 | 15.17 | 75.59 |
| 2012 | Income: Q1 | 2,523 | 85.98 | 5.53 | 8.49 |
| 2012 | Income: Q2 | 2,725 | 77.23 | 9.52 | 13.25 |
| 2012 | Income: Q3 | 2,656 | 59.67 | 15.82 | 24.51 |
| 2012 | Income: Q4 | 2,716 | 29.95 | 18.37 | 51.67 |
| 2013 | Education: No formal schooling/private school/literacy class | 1,610 | 96.36 | 2.77 | 0.87 |
| 2013 | Education: Primary | 2,445 | 88.16 | 7.85 | 3.99 |
| 2013 | Education: Junior secondary | 3,316 | 54.21 | 22.00 | 23.79 |
| 2013 | Education: Upper secondary / vocational | 2,178 | 28.46 | 22.63 | 48.91 |
| 2013 | Education: Tertiary | 1,858 | 7.22 | 11.66 | 81.11 |
| 2013 | Income: Q1 | 2,658 | 71.76 | 10.97 | 17.27 |
| 2013 | Income: Q2 | 2,536 | 70.96 | 12.18 | 16.86 |
| 2013 | Income: Q3 | 2,677 | 48.24 | 20.17 | 31.59 |
| 2013 | Income: Q4 | 2,347 | 24.49 | 19.15 | 56.36 |
| 2015 | Education: No formal schooling/private school/literacy class | 1,560 | 95.61 | 3.24 | 1.15 |
| 2015 | Education: Primary | 2,536 | 80.59 | 12.96 | 6.45 |
| 2015 | Education: Junior secondary | 3,074 | 45.38 | 19.22 | 35.40 |
| 2015 | Education: Upper secondary / vocational | 1,959 | 22.92 | 17.63 | 59.45 |
| 2015 | Education: Tertiary | 1,762 | 4.72 | 8.15 | 87.13 |
| 2015 | Income: Q1 | 2,907 | 62.10 | 10.11 | 27.79 |
| 2015 | Income: Q2 | 2,238 | 66.00 | 14.07 | 19.93 |
| 2015 | Income: Q3 | 2,807 | 43.18 | 17.58 | 39.24 |
| 2015 | Income: Q4 | 2,365 | 19.43 | 16.19 | 64.38 |
| 2017 | Education: No formal schooling/private school/literacy class | 1,617 | 79.41 | 8.08 | 12.51 |
| 2017 | Education: Primary | 2,726 | 53.17 | 20.41 | 26.42 |
| 2017 | Education: Junior secondary | 3,510 | 18.66 | 17.62 | 63.73 |
| 2017 | Education: Upper secondary / vocational | 2,233 | 6.45 | 9.96 | 83.59 |
| 2017 | Education: Tertiary | 2,467 | 1.13 | 3.36 | 95.51 |
| 2017 | Income: Q1 | 2,853 | 26.70 | 11.88 | 61.42 |
| 2017 | Income: Q2 | 3,327 | 33.05 | 17.73 | 49.22 |
| 2017 | Income: Q3 | 2,697 | 15.07 | 13.79 | 71.14 |
| 2017 | Income: Q4 | 3,014 | 7.22 | 9.93 | 82.85 |
| 2018 | Education: No formal schooling/private school/literacy class | 1,939 | 63.85 | 12.03 | 24.12 |
| 2018 | Education: Primary | 2,722 | 42.52 | 23.52 | 33.96 |
| 2018 | Education: Junior secondary | 3,368 | 14.76 | 18.19 | 67.05 |
| 2018 | Education: Upper secondary / vocational | 2,329 | 5.92 | 12.36 | 81.72 |
| 2018 | Education: Tertiary | 2,393 | 0.93 | 4.05 | 95.02 |
| 2018 | Income: Q1 | 2,955 | 24.25 | 14.50 | 61.25 |
| 2018 | Income: Q2 | 3,224 | 28.32 | 20.70 | 50.98 |
| 2018 | Income: Q3 | 2,870 | 10.98 | 14.91 | 74.11 |
| 2018 | Income: Q4 | 3,051 | 3.44 | 8.93 | 87.63 |
| 2021 | Education: No formal schooling/private school/literacy class | 904 | 46.43 | 17.51 | 36.06 |
| 2021 | Education: Primary | 1,750 | 27.42 | 21.83 | 50.75 |
| 2021 | Education: Junior secondary | 2,307 | 8.90 | 13.90 | 77.20 |
| 2021 | Education: Upper secondary / vocational | 1,488 | 2.47 | 6.44 | 91.08 |
| 2021 | Education: Tertiary | 1,671 | 0.60 | 3.61 | 95.80 |
| 2021 | Income: Q1 | 2,414 | 18.87 | 15.53 | 65.60 |
| 2021 | Income: Q2 | 2,048 | 13.37 | 18.59 | 68.04 |
| 2021 | Income: Q3 | 1,267 | 10.17 | 12.17 | 77.66 |
| 2021 | Income: Q4 | 1,597 | 3.05 | 5.83 | 91.12 |
| 2023 | Education: No formal schooling/private school/literacy class | 634 | 45.68 | 22.80 | 31.52 |
| 2023 | Education: Primary | 1,305 | 27.07 | 26.05 | 46.88 |
| 2023 | Education: Junior secondary | 1,616 | 9.98 | 15.11 | 74.92 |
| 2023 | Education: Upper secondary / vocational | 1,037 | 6.04 | 9.87 | 84.09 |
| 2023 | Education: Tertiary | 985 | 1.03 | 3.90 | 95.07 |
| 2023 | Income: Q1 | 1,405 | 24.30 | 17.36 | 58.35 |
| 2023 | Income: Q2 | 1,403 | 18.82 | 19.48 | 61.70 |
| 2023 | Income: Q3 | 1,396 | 7.80 | 12.28 | 79.92 |
| 2023 | Income: Q4 | 1,151 | 3.22 | 7.63 | 89.15 |

Counts are unweighted valid-group records; percentages are official-weighted and sum to 100% within each row apart from rounding. Income quartiles use D18 weighted personal-income midpoint ranks: Q1 ≤0.25, Q2 >0.25–0.50, Q3 >0.50–0.75, Q4 >0.75. Ties are retained.

# Supplementary Table S6. Binary standardized exercise associations and sensitivity thresholds

Within each A30 wave, the core survey-weighted logistic model includes binary internet use, a four-degree-of-freedom natural spline for age, sex, ethnicity, five education levels, agricultural versus other hukou, and province indicators. No exposure interaction is included. Each respondent is predicted once under each exposure value while retaining their observed covariates; the official-weighted average prediction difference gives the wave-specific standardized difference. Province is a geographic-cluster proxy, not a recovered official primary sampling unit.

## S6A. Nine-wave binary differences, probability ratios and province counts

| **Year** | **Nonfrequent n** | **Frequent n** | **Provinces G** | **CI df** | **Difference, pp [95% CI]** | **Probability ratio [95% CI]** |
| --- | --- | --- | --- | --- | --- | --- |
| 2010 | 7,897 | 1,410 | 31 | 30 | 2.12 [−0.49, 4.73] | 1.117 [0.976, 1.280] |
| 2011 | 3,798 | 639 | 26 | 25 | 6.51 [2.72, 10.30] | 1.286 [1.117, 1.481] |
| 2012 | 7,564 | 1,781 | 29 | 28 | 5.78 [2.84, 8.72] | 1.294 [1.162, 1.440] |
| 2013 | 7,163 | 1,814 | 28 | 27 | 4.04 [1.04, 7.04] | 1.237 [1.061, 1.442] |
| 2015 | 6,240 | 2,184 | 28 | 27 | 6.85 [1.86, 11.84] | 1.261 [1.054, 1.509] |
| 2017 | 5,834 | 4,044 | 28 | 27 | 10.09 [7.81, 12.38] | 1.428 [1.285, 1.586] |
| 2018 | 5,640 | 4,426 | 28 | 27 | 8.35 [4.85, 11.85] | 1.312 [1.171, 1.470] |
| 2021 | 2,727 | 3,536 | 19 | 18 | 5.04 [1.37, 8.71] | 1.156 [1.038, 1.287] |
| 2023 | 1,638 | 2,883 | 16 | 15 | 5.16 [−0.05, 10.38] | 1.193 [1.007, 1.413] |

Core-adjusted A30 estimates standardized to each wave’s weighted A30 population. Variance includes model-coefficient uncertainty, empirical-target uncertainty and their covariance. Pointwise t intervals use G−1 degrees of freedom; they are not simultaneous intervals or equivalence tests. Differences are Frequent minus Nonfrequent; ratios are Frequent divided by Nonfrequent. Ratio intervals use the log-scale method in S7. The 2023 difference interval includes 0 whereas its ratio interval excludes 1, reflecting scale-dependent Wald approximation near the null.

The 2010 frequent category has no Tibet observations, requiring prediction for that province in the wave target. Binary and three-category models are fitted separately; regrouping the three-category results is not an exact decomposition of the binary estimate. Difference intervals crossing zero are retained for 2010 and 2023. The 2023 probability-ratio interval, calculated on the log scale and back-transformed, instead excludes 1; the two nonlinear Wald approximations can differ near the null because they use different scales. Pointwise uncertainty does not test equivalence between waves or between categories.

For each wave, let A denote frequent (1) versus nonfrequent (0) Internet use, Y regular exercise, C the E1 covariates, and U unmeasured confounders. We assume consistency, positivity, and sufficient adjustment by C and U. The strong null sets the mean causal risk ratio to one within every C stratum. Let R_AU(c) be the largest ratio of the distributions of U between A=1 and A=0 given C=c, and R_UY(c) the largest ratio of exercise risks across U within either exposure group at C=c. We impose a common upper bound s≥1 on both parameters for every c [SR9].

$\begin{aligned} R_{\mathrm{AU}}(c)=\sup_{u}\frac{f(u|A=1,c)}{f(u|A=0,c)}\leq s \\ R_{\mathrm{UY}}(c)=\max_{a}\frac{\sup_{u}P(Y=1|a,c,u)}{\inf_{u}P(Y=1|a,c,u)}\leq s \end{aligned}$

Writing p_a(c)=P(Y=1|A=a,C=c), the original conditional bound [SR9] under this strong null gives the first inequality below. Averaging both sides over the same officially weighted A30 distribution F_w(c) preserves the inequality. Inverting B(s) gives the E-value-formula threshold [SR10]; homogeneity of causal risk ratios under alternatives is not assumed.

$\begin{aligned} p_{1}(c)\leq B(s)p_{0}(c),B(s)=\frac{s^{2}}{2s-1} \\ R=\frac{\int p_{1}(c)dF_{w}(c)}{\int p_{0}(c)dF_{w}(c)}\leq B(s) \end{aligned}$

$s\geq E(R)=R+\sqrt{R(R-1)},R\geq1$

## S6B. E-value-formula necessary thresholds under the stratum-specific strong null

| **Year** | **Point-estimate threshold** | **Threshold for CI limit closest to null** |
| --- | --- | --- |
| 2010 | 1.480 | 1.000 |
| 2011 | 1.893 | 1.478 |
| 2012 | 1.910 | 1.596 |
| 2013 | 1.779 | 1.316 |
| 2015 | 1.836 | 1.293 |
| 2017 | 2.209 | 1.891 |
| 2018 | 1.952 | 1.619 |
| 2021 | 1.581 | 1.237 |
| 2023 | 1.673 | 1.092 |

A30 E1 binary Frequent/Nonfrequent comparisons only; PRs and their original pointwise log-delta-t 95% CIs (df=G−1) are in S6A. The closest-limit threshold equals 1 if the PR CI includes 1; otherwise the lower limit is substituted into E(R). It is not a confidence interval for the threshold. The bound is necessary, not sufficient, and is not established as sharp for the complete observed data. It neither requires both association parameters to exceed the threshold nor states that confounding of this strength would remove statistical significance. The 2023 PD CI still includes zero.

These calculations are conditional on the fitted probabilities, including predictions outside observed exposure-group support: Frequent users in 2010 had no Tibet observations (0.20% of the weighted A30 target), and their observed age range in 2011 was 30–73 years (1.55% of the target lay outside it). The thresholds do not correct model misspecification, extrapolation, measurement error, or reverse causation, and do not quantify the exact minimum confounding required to eliminate a marginal average causal effect.

# Supplementary Table S7. Three-category standardized exercise probabilities and contrasts

The three- and five-category models use the same A30 records, weights, core adjustment variables, four-degree-of-freedom age spline and empirical wave target as the binary analysis; exposure is entered as a factor. For category k, P̂ₖ = Σᵢwᵢm̂(k,Xᵢ)/Σᵢwᵢ. The joint influence for P̂ₖ combines the model-coefficient contribution with the empirical-target contribution [wᵢ/Σw][m̂(k,Xᵢ)−P̂ₖ]. Their sum is aggregated by the province-cluster proxy to estimate the complete category-probability covariance matrix, including model–target cross-covariance. For contrast vector c, Δ̂ = cᵀP̂ and Var(Δ̂) = cᵀV̂c.

Probability intervals are computed on the logit scale using t with G−1 degrees of freedom and transformed back to the probability scale. Difference intervals use the corresponding t interval on the difference scale. Each probability is the predicted exercise outcome under a specified internet-use category; the probabilities are not internet-use shares and do not sum to 100%. All intervals are pointwise. For binary and three-category contrasts, R = p_a/p_b. With V the full joint covariance defined above, SE(log R) = [V_aa/p_a² + V_bb/p_b² − 2V_ab/(p_a p_b)]½; the 95% interval is exp[log R ± t_(G−1,0.975) × SE(log R)]. Ratios use unrounded probabilities and covariances. They are not conditional odds ratios or longitudinal incidence risk ratios.

## S7A. Three-category exercise probabilities

| **Year** | **Never % [95% CI]** | **Low % [95% CI]** | **Frequent % [95% CI]** |
| --- | --- | --- | --- |
| 2010 | 16.99 [13.45, 21.23] | 21.48 [17.77, 25.71] | 21.28 [17.42, 25.74]* |
| 2011 | 20.95 [16.16, 26.70] | 29.07 [22.94, 36.08] | 31.39 [24.75, 38.90]‡ |
| 2012 | 17.56 [13.75, 22.16] | 25.89 [20.73, 31.81] | 27.42 [21.71, 33.99] |
| 2013 | 16.54 [12.65, 21.32] | 18.29 [13.72, 23.96] | 21.49 [16.92, 26.88] |
| 2015 | 23.78 [19.23, 29.02] | 32.00 [26.64, 37.88] | 34.56 [30.51, 38.85] |
| 2017 | 20.71 [16.32, 25.91] | 27.29 [22.71, 32.40] | 34.35 [30.32, 38.63] |
| 2018 | 24.48 [20.21, 29.32] | 28.85 [23.84, 34.43] | 35.45 [30.49, 40.75] |
| 2021 | 29.65 [25.63, 34.03]§ | 33.92 [28.49, 39.81] | 37.47 [33.98, 41.10] |
| 2023 | 25.80 [20.89, 31.41]† | 27.34 [21.69, 33.83] | 31.95 [25.14, 39.63] |

Each category is standardized to the same weighted A30 target within the wave. These are separate outcome probabilities and do not sum to 100%. Intervals are pointwise. Support markers: * 2010 Frequent lacked Tibet observations (0.20% of the weighted A30 target); ‡ 2011 Frequent ages 30–73 (1.55% outside); § 2021 Never ages 31–74 (3.13% outside); † 2023 Never ages 36–74 (14.43% outside). A marker on a difference identifies the involved category. Joint-cell gaps also exist; these percentages must not be added.

## S7B. All three category contrasts in every wave

| **Year** | **Comparison** | **Difference, pp [95% CI]** | **Probability ratio [95% CI]** |
| --- | --- | --- | --- |
| 2010 | Frequent / Never | 4.29 [1.17, 7.42]* | 1.253 [1.060, 1.480]* |
| 2010 | Low / Never | 4.49 [1.80, 7.18] | 1.264 [1.091, 1.465] |
| 2010 | Frequent / Low | −0.19 [−2.93, 2.54]* | 0.991 [0.872, 1.126]* |
| 2011 | Frequent / Never | 10.45 [5.98, 14.91]‡ | 1.499 [1.276, 1.760]‡ |
| 2011 | Low / Never | 8.13 [4.27, 11.98] | 1.388 [1.196, 1.611] |
| 2011 | Frequent / Low | 2.32 [−1.60, 6.24]‡ | 1.080 [0.950, 1.228]‡ |
| 2012 | Frequent / Never | 9.86 [5.85, 13.87] | 1.561 [1.331, 1.832] |
| 2012 | Low / Never | 8.32 [4.15, 12.50] | 1.474 [1.223, 1.777] |
| 2012 | Frequent / Low | 1.54 [−1.31, 4.38] | 1.059 [0.954, 1.176] |
| 2013 | Frequent / Never | 4.95 [1.96, 7.94] | 1.299 [1.110, 1.521] |
| 2013 | Low / Never | 1.75 [−1.83, 5.34] | 1.106 [0.904, 1.353] |
| 2013 | Frequent / Low | 3.19 [−0.83, 7.21] | 1.175 [0.954, 1.446] |
| 2015 | Frequent / Never | 10.78 [5.71, 15.85] | 1.453 [1.196, 1.766] |
| 2015 | Low / Never | 8.21 [4.76, 11.67] | 1.345 [1.186, 1.526] |
| 2015 | Frequent / Low | 2.56 [−3.08, 8.21] | 1.080 [0.909, 1.284] |
| 2017 | Frequent / Never | 13.65 [10.82, 16.47] | 1.659 [1.427, 1.929] |
| 2017 | Low / Never | 6.58 [3.03, 10.12] | 1.318 [1.124, 1.544] |
| 2017 | Frequent / Low | 7.07 [4.11, 10.03] | 1.259 [1.128, 1.405] |
| 2018 | Frequent / Never | 10.97 [6.98, 14.96] | 1.448 [1.259, 1.666] |
| 2018 | Low / Never | 4.37 [−0.10, 8.83] | 1.178 [0.999, 1.391] |
| 2018 | Frequent / Low | 6.61 [2.47, 10.74] | 1.229 [1.074, 1.406] |
| 2021 | Frequent / Never | 7.82 [2.59, 13.05]§ | 1.264 [1.074, 1.487]§ |
| 2021 | Low / Never | 4.27 [−3.03, 11.57]§ | 1.144 [0.912, 1.435]§ |
| 2021 | Frequent / Low | 3.55 [−1.42, 8.52] | 1.105 [0.955, 1.278] |
| 2023 | Frequent / Never | 6.15 [0.81, 11.50]† | 1.238 [1.041, 1.474]† |
| 2023 | Low / Never | 1.54 [−2.10, 5.19]† | 1.060 [0.926, 1.213]† |
| 2023 | Frequent / Low | 4.61 [−0.95, 10.17] | 1.169 [0.972, 1.404] |

Differences subtract the second named group from the first (percentage points); ratios divide the first group’s probability by the second’s (dimensionless). Both show 95% CI. Models and targets are the same as Supplementary Table S7A. Binary and three-category models were fitted separately; their differences are not an exact decomposition of the binary estimate. Support markers: * 2010 Frequent lacked Tibet observations (0.20% of the weighted A30 target); ‡ 2011 Frequent ages 30–73 (1.55% outside); § 2021 Never ages 31–74 (3.13% outside); † 2023 Never ages 36–74 (14.43% outside). A marker on a contrast identifies the involved category on both scales. Joint-cell gaps also exist; these percentages must not be added.

# Supplementary Table S8. Five-category exercise probabilities and adjacent-category contrasts

The five responses are entered as factor levels: 1 never, 2 rarely, 3 sometimes, 4 often, and 5 always/very frequently. All 45 category-specific probabilities are reported. To describe differences between adjacent responses, the same four contrasts (2−1, 3−2, 4−3 and 5−4) are displayed in every wave, regardless of direction or whether the interval crosses zero. No equal spacing between responses is assumed. Estimates use each wave’s A30 target and the joint-covariance method in S7; all intervals are pointwise.

## S8A. All 45 standardized exercise probabilities

| **Year** | **1 % [95% CI]** | **2 % [95% CI]** | **3 % [95% CI]** | **4 % [95% CI]** | **5 % [95% CI]** |
| --- | --- | --- | --- | --- | --- |
| 2010 | 16.95 [13.41, 21.19] | 20.59 [16.36, 25.59] | 22.45 [18.20, 27.35] | 20.25 [16.09, 25.15] | 22.93 [18.44, 28.13] |
| 2011 | 20.97 [16.18, 26.73] | 28.65 [22.05, 36.31] | 29.50 [22.67, 37.40] | 34.40 [27.33, 42.25] | 28.48 [21.30, 36.95] |
| 2012 | 17.51 [13.71, 22.11] | 23.64 [17.64, 30.92] | 28.06 [22.70, 34.14] | 28.36 [22.46, 35.11] | 26.93 [20.62, 34.33] |
| 2013 | 16.56 [12.63, 21.41] | 18.65 [13.39, 25.37] | 17.92 [13.38, 23.58] | 21.89 [15.96, 29.26] | 20.96 [16.38, 26.40] |
| 2015 | 23.78 [19.23, 29.03] | 30.98 [25.30, 37.29] | 33.07 [27.40, 39.27] | 35.29 [31.66, 39.10] | 33.90 [28.85, 39.35] |
| 2017 | 20.61 [16.15, 25.92] | 28.55 [22.13, 35.97] | 26.40 [22.22, 31.06] | 33.08 [28.12, 38.46] | 35.54 [31.36, 39.95] |
| 2018 | 24.40 [20.21, 29.16] | 29.59 [22.92, 37.26] | 28.25 [23.41, 33.66] | 34.48 [29.75, 39.54] | 36.47 [30.84, 42.49] |
| 2021 | 29.63 [25.59, 34.02] | 34.14 [26.65, 42.52] | 33.74 [27.42, 40.71] | 36.93 [31.95, 42.21] | 37.80 [34.07, 41.68] |
| 2023 | 25.47 [20.56, 31.11] | 25.56 [20.33, 31.60] | 28.40 [20.83, 37.42] | 29.20 [22.29, 37.23] | 33.73 [26.96, 41.24] |

Response 1 in 2023 has observed ages 36–74; 14.43% of the weighted A30 target lies outside this range. Other category-specific age and joint-cell gaps are detailed in S9. Finite estimates do not remove those gaps.

## S8B. Adjacent-category differences in all nine waves

| **Year** | **2−1** | **3−2** | **4−3** | **5−4** |
| --- | --- | --- | --- | --- |
| 2010 | 3.64 [0.36, 6.92] | 1.86 [−2.87, 6.58] | −2.20 [−6.14, 1.73] | 2.68 [−1.75, 7.12] |
| 2011 | 7.68 [3.01, 12.35] | 0.85 [−5.32, 7.02] | 4.90 [−1.33, 11.14] | −5.92 [−10.96, −0.89] |
| 2012 | 6.13 [0.69, 11.56] | 4.42 [−1.09, 9.94] | 0.30 [−2.95, 3.54] | −1.43 [−6.40, 3.53] |
| 2013 | 2.09 [−2.29, 6.47] | −0.73 [−4.96, 3.50] | 3.97 [−0.24, 8.17] | −0.93 [−7.44, 5.58] |
| 2015 | 7.19 [3.61, 10.78] | 2.09 [−2.01, 6.19] | 2.22 [−3.45, 7.89] | −1.39 [−4.82, 2.04] |
| 2017 | 7.94 [2.74, 13.14] | −2.14 [−7.66, 3.37] | 6.68 [2.37, 10.99] | 2.46 [−2.44, 7.35] |
| 2018 | 5.18 [−1.00, 11.37] | −1.34 [−7.35, 4.68] | 6.23 [1.08, 11.38] | 1.99 [−1.67, 5.65] |
| 2021 | 4.51 [−3.49, 12.51] | −0.40 [−9.53, 8.73] | 3.19 [−2.93, 9.31] | 0.87 [−4.31, 6.04] |
| 2023 | 0.09 [−4.95, 5.13] | 2.84 [−5.01, 10.69] | 0.80 [−5.20, 6.80] | 4.52 [1.45, 7.59] |

Percentage-point differences [95% CI], using the same models, targets and province-proxy t inference as S8A. The 36 displayed contrasts follow one rule across all nine waves; negative estimates and intervals crossing zero are retained. Observed-support gaps are reported in S9.

# Supplementary Table S9. Observed comparison support and model diagnostics

The standardization target comprises all respondents aged 30–74 in each year’s A30 association sample, with composition defined by that year’s official survey weights. Observed support assesses whether each internet-use category contains respondents with the ages and social characteristics represented in this target; it is distinct from numerical model estimability. Category counts, Kish ESS and observed age ranges are reported for every wave. Marginal cross-tabs examine 5-year age bands, sex, ethnicity, education, hukou and province. The joint diagnostic uses the observed target combinations of 5-year age band × sex × education × hukou. For each category, unsupported target weight is the sum of official weights in target cells without a record from that category divided by total A30 target weight. It is a descriptive measure of model reliance, not an estimated probability of bias. These gaps describe absent comparison-group observations at target profiles, not missing information among A30 respondents.

## S9A. Category size and observed age support

| **Year** | **Scheme/category** | **n** | **Kish ESS** | **Observed ages** | **Target outside %** |
| --- | --- | --- | --- | --- | --- |
| 2010 | Three/Never | 6,707 | 5634.44 | 30–74 | 0.00 |
| 2010 | Three/Low | 1,190 | 1039.89 | 30–74 | 0.00 |
| 2010 | Three/Frequent | 1,410 | 1237.17 | 30–74 | 0.00 |
| 2011 | Three/Never | 3,208 | 2293.52 | 30–74 | 0.00 |
| 2011 | Three/Low | 590 | 424.67 | 30–74 | 0.00 |
| 2011 | Three/Frequent | 639 | 456.45 | 30–73 | 1.55 |
| 2012 | Three/Never | 6,226 | 4406.59 | 30–74 | 0.00 |
| 2012 | Three/Low | 1,338 | 998.84 | 30–74 | 0.00 |
| 2012 | Three/Frequent | 1,781 | 1310.05 | 30–74 | 0.00 |
| 2013 | Three/Never | 5,641 | 3709.98 | 30–74 | 0.00 |
| 2013 | Three/Low | 1,522 | 1063.83 | 30–74 | 0.00 |
| 2013 | Three/Frequent | 1,814 | 1256.34 | 30–74 | 0.00 |
| 2015 | Three/Never | 4,873 | 3465.25 | 30–74 | 0.00 |
| 2015 | Three/Low | 1,367 | 1016.89 | 30–74 | 0.00 |
| 2015 | Three/Frequent | 2,184 | 1517.23 | 30–74 | 0.00 |
| 2017 | Three/Never | 4,381 | 2128.13 | 30–74 | 0.00 |
| 2017 | Three/Low | 1,453 | 815.42 | 30–74 | 0.00 |
| 2017 | Three/Frequent | 4,044 | 2253.95 | 30–74 | 0.00 |
| 2018 | Three/Never | 3,872 | 1663.16 | 30–74 | 0.00 |
| 2018 | Three/Low | 1,768 | 763.17 | 30–74 | 0.00 |
| 2018 | Three/Frequent | 4,426 | 2087.74 | 30–74 | 0.00 |
| 2021 | Three/Never | 1,707 | 690.21 | 31–74 | 3.13 |
| 2021 | Three/Low | 1,020 | 477.21 | 30–74 | 0.00 |
| 2021 | Three/Frequent | 3,536 | 1614.78 | 30–74 | 0.00 |
| 2023 | Three/Never | 768 | 556.61 | 36–74 | 14.43 |
| 2023 | Three/Low | 870 | 613.71 | 30–74 | 0.00 |
| 2023 | Three/Frequent | 2,883 | 2009.73 | 30–74 | 0.00 |
| 2010 | Five/1 | 6,707 | 5634.44 | 30–74 | 0.00 |
| 2010 | Five/2 | 615 | 535.11 | 30–74 | 0.00 |
| 2010 | Five/3 | 575 | 505.06 | 30–74 | 0.00 |
| 2010 | Five/4 | 794 | 704.12 | 30–74 | 0.00 |
| 2010 | Five/5 | 616 | 533.09 | 30–70 | 3.39 |
| 2011 | Five/1 | 3,208 | 2293.52 | 30–74 | 0.00 |
| 2011 | Five/2 | 315 | 219.36 | 30–73 | 1.55 |
| 2011 | Five/3 | 275 | 206.64 | 30–74 | 0.00 |
| 2011 | Five/4 | 324 | 226.45 | 30–69 | 7.72 |
| 2011 | Five/5 | 315 | 230.20 | 30–73 | 1.55 |
| 2012 | Five/1 | 6,226 | 4406.59 | 30–74 | 0.00 |
| 2012 | Five/2 | 646 | 487.10 | 30–74 | 0.00 |
| 2012 | Five/3 | 692 | 511.88 | 30–74 | 0.00 |
| 2012 | Five/4 | 852 | 639.82 | 30–74 | 0.00 |
| 2012 | Five/5 | 929 | 675.38 | 30–74 | 0.00 |
| 2013 | Five/1 | 5,641 | 3709.98 | 30–74 | 0.00 |
| 2013 | Five/2 | 776 | 556.46 | 30–74 | 0.00 |
| 2013 | Five/3 | 746 | 507.99 | 30–74 | 0.00 |
| 2013 | Five/4 | 925 | 616.52 | 30–74 | 0.00 |
| 2013 | Five/5 | 889 | 642.57 | 30–74 | 0.00 |
| 2015 | Five/1 | 4,873 | 3465.25 | 30–74 | 0.00 |
| 2015 | Five/2 | 683 | 510.80 | 30–74 | 0.00 |
| 2015 | Five/3 | 684 | 506.29 | 30–74 | 0.00 |
| 2015 | Five/4 | 1,059 | 778.35 | 30–74 | 0.00 |
| 2015 | Five/5 | 1,125 | 747.66 | 30–74 | 0.00 |
| 2017 | Five/1 | 4,381 | 2128.13 | 30–74 | 0.00 |
| 2017 | Five/2 | 632 | 356.88 | 30–74 | 0.00 |
| 2017 | Five/3 | 821 | 467.05 | 30–74 | 0.00 |
| 2017 | Five/4 | 1,813 | 1013.22 | 30–74 | 0.00 |
| 2017 | Five/5 | 2,231 | 1240.99 | 30–74 | 0.00 |
| 2018 | Five/1 | 3,872 | 1663.16 | 30–74 | 0.00 |
| 2018 | Five/2 | 788 | 336.49 | 30–74 | 0.00 |
| 2018 | Five/3 | 980 | 428.28 | 30–74 | 0.00 |
| 2018 | Five/4 | 2,153 | 1026.42 | 30–74 | 0.00 |
| 2018 | Five/5 | 2,273 | 1061.33 | 30–74 | 0.00 |
| 2021 | Five/1 | 1,707 | 690.21 | 31–74 | 3.13 |
| 2021 | Five/2 | 455 | 207.88 | 30–74 | 0.00 |
| 2021 | Five/3 | 565 | 272.63 | 30–74 | 0.00 |
| 2021 | Five/4 | 1,344 | 613.43 | 30–74 | 0.00 |
| 2021 | Five/5 | 2,192 | 1002.49 | 30–74 | 0.00 |
| 2023 | Five/1 | 768 | 556.61 | 36–74 | 14.43 |
| 2023 | Five/2 | 389 | 273.34 | 30–74 | 0.00 |
| 2023 | Five/3 | 481 | 340.58 | 33–74 | 5.87 |
| 2023 | Five/4 | 1,097 | 757.30 | 30–74 | 0.00 |
| 2023 | Five/5 | 1,786 | 1252.58 | 30–74 | 0.00 |

The final column uses the total official weight of all A30 respondents in that wave as its denominator. In 2023, never users were observed only at ages 36–74; ages 30–35 account for 14.43% of the full weighted A30 target. Five-category codes follow S8.

## S9B. Nonzero marginal support gaps

| **Year** | **Scheme/category** | **Observed gap** | **Weighted target %** |
| --- | --- | --- | --- |
| 2010 | Three/Frequent | Empty province level: Tibet | 0.20 |
| 2011 | Three/Frequent | Observed age range: Observed 30–73 years | 1.55 |
| 2021 | Three/Never | Observed age range: Observed 31–74 years | 3.13 |
| 2023 | Three/Never | Empty 5-year age band: 30-34 | 11.61 |
| 2023 | Three/Never | Observed age range: Observed 36–74 years | 14.43 |
| 2010 | Five/2 | Province: Inner Mongolia | 0.83 |
| 2010 | Five/4 | Province: Tibet | 0.20 |
| 2010 | Five/5 | Province: Shaanxi; Tibet; Chongqing | 6.71 |
| 2011 | Five/3 | Education: No formal schooling/private school/literacy class | 14.51 |
| 2011 | Five/3 | Province: Yunnan | 3.51 |
| 2011 | Five/4 | 5-year age band: 70-74 | 7.72 |
| 2011 | Five/5 | Province: Yunnan | 3.51 |
| 2023 | Five/1 | 5-year age band: 30-34 | 11.61 |

Percentages refer to the whole weighted A30 target. The 2023 empty 30–34 age band is part of the 30–35 age-range gap (14.43%) and cannot be added to it. Observed age-range gaps for every category are in S9A. Marginal and joint gaps overlap; these percentages do not estimate a probability of bias.

## S9C. Joint age sex education and hukou support gaps

| **Year** | **Never** | **Low** | **Frequent** | **1** | **2** | **3** | **4** | **5** |
| --- | --- | --- | --- | --- | --- | --- | --- | --- |
| 2010 | 4 (0.13) | 41 (9.41) | 69 (24.47) | 4 (0.13) | 49 (11.32) | 80 (33.90) | 83 (36.35) | 90 (36.36) |
| 2011 | 5 (0.44) | 70 (26.92) | 77 (34.91) | 5 (0.44) | 80 (29.23) | 91 (40.33) | 96 (47.59) | 91 (43.56) |
| 2012 | 8 (0.33) | 35 (9.98) | 49 (23.88) | 8 (0.33) | 50 (14.31) | 69 (29.84) | 68 (35.14) | 69 (29.60) |
| 2013 | 6 (1.71) | 25 (4.52) | 46 (9.67) | 6 (1.71) | 39 (7.33) | 55 (17.86) | 63 (16.32) | 73 (23.12) |
| 2015 | 19 (5.20) | 25 (2.20) | 42 (12.88) | 19 (5.20) | 38 (5.10) | 46 (8.79) | 59 (19.38) | 59 (18.77) |
| 2017 | 29 (11.84) | 21 (1.75) | 18 (2.06) | 29 (11.84) | 49 (11.13) | 36 (3.49) | 30 (2.72) | 32 (5.39) |
| 2018 | 28 (13.61) | 16 (1.69) | 4 (0.02) | 28 (13.61) | 39 (7.14) | 26 (3.78) | 9 (0.48) | 25 (3.57) |
| 2021 | 65 (29.69) | 33 (5.91) | 4 (0.07) | 65 (29.69) | 61 (18.49) | 49 (12.00) | 19 (0.89) | 18 (0.66) |
| 2023 | 80 (37.25) | 31 (10.08) | 4 (0.08) | 80 (37.25) | 65 (25.75) | 50 (18.27) | 21 (1.41) | 10 (1.47) |

The first three category columns use the three-category model, and columns 1–5 use the five-category model. Each cell gives unsupported observed target combinations (weighted target %). Combinations use 5-year age bands, sex, education and hukou. Percentages overlap and must not be added. These are support diagnostics, not additional exclusion rules.

## S9D. Summary diagnostics for all 18 category models

| **Year** | **Scheme** | **Rank / parameters** | **Observed fitted range** |
| --- | --- | --- | --- |
| 2010 | Three | 44/44 | 0.00551–0.87982 |
| 2011 | Three | 39/39 | 0.00782–0.85713 |
| 2012 | Three | 42/42 | 0.00146–0.82898 |
| 2013 | Three | 41/41 | 0.00472–0.78827 |
| 2015 | Three | 41/41 | 0.02403–0.86217 |
| 2017 | Three | 41/41 | 0.02768–0.83522 |
| 2018 | Three | 41/41 | 0.01693–0.81590 |
| 2021 | Three | 32/32 | 0.08589–0.77738 |
| 2023 | Three | 29/29 | 0.02861–0.79098 |
| 2010 | Five | 46/46 | 0.00546–0.88510 |
| 2011 | Five | 41/41 | 0.00797–0.85383 |
| 2012 | Five | 44/44 | 0.00147–0.84601 |
| 2013 | Five | 43/43 | 0.00473–0.78407 |
| 2015 | Five | 43/43 | 0.02409–0.86689 |
| 2017 | Five | 43/43 | 0.02796–0.83958 |
| 2018 | Five | 43/43 | 0.01655–0.80921 |
| 2021 | Five | 34/34 | 0.08547–0.77993 |
| 2023 | Five | 31/31 | 0.02497–0.78397 |

All 18 models converged with full-rank matrices, finite coefficients and no fitting warnings. No observed fitted probability was below 10⁻⁶ or above 1−10⁻⁶. Province counts and G−1 interval degrees of freedom are listed in S6A. Numerical convergence does not establish support at every target profile.

# Supplementary Table S10. Adjustment on identical records and missing-data sensitivities

The contrast is frequent minus nonfrequent general internet use, and the outcome is leisure exercise daily or several times per week. C30 comprises 64,000 of the 71,218 A30 records with complete marital status, employment and household income; 7,218 records are excluded. Within each wave, E0 (unadjusted), E1 (core adjusted) and E2 (extended adjusted) use identical C30 records, official weights and the weighted C30 standardization target. E1 adjusts for a four-degree-of-freedom age natural spline, sex, ethnicity, five-category education, agricultural hukou and province. E2 adds marital status, employment and the survey-weighted household-income rank within that wave's C30 sample; this differs from the D18 personal-income reference used for descriptive analyses. Joint influence variance includes model uncertainty, empirical-target uncertainty and their covariance. Wave-specific intervals use a t reference with G−1 degrees of freedom, where G is the number of province proxies (Supplementary Table S13A).

## S10A. Wave-specific E0, E1, and E2 differences on C30

| **Year** | **C30 n** | **E0 [95% CI]** | **E1 [95% CI]** | **E2 [95% CI]** |
| --- | --- | --- | --- | --- |
| 2010 | 8,327 | 15.77 [11.54, 20.00] | 2.14 [−0.13, 4.41] | 2.01 [−0.03, 4.06] |
| 2011 | 4,018 | 19.01 [11.43, 26.59] | 6.62 [2.58, 10.66] | 6.15 [1.85, 10.46] |
| 2012 | 8,427 | 17.96 [12.03, 23.89] | 5.56 [2.28, 8.83] | 5.13 [2.09, 8.17] |
| 2013 | 7,907 | 10.04 [5.23, 14.85] | 3.78 [1.44, 6.13] | 3.59 [1.42, 5.76] |
| 2015 | 7,798 | 12.41 [8.57, 16.25] | 7.01 [1.85, 12.18] | 6.73 [1.55, 11.90] |
| 2017 | 9,082 | 15.15 [12.22, 18.08] | 11.03 [8.81, 13.25] | 9.53 [7.09, 11.97] |
| 2018 | 9,125 | 11.51 [8.07, 14.94] | 8.20 [4.40, 12.00] | 6.51 [2.51, 10.52] |
| 2021 | 5,129 | 4.99 [1.07, 8.92] | 4.97 [0.66, 9.28] | 4.15 [−0.25, 8.54] |
| 2023 | 4,187 | 7.06 [2.59, 11.54] | 4.54 [−0.95, 10.04] | 4.03 [−1.33, 9.40] |

Differences and 95% intervals are in percentage points. C30 is the common denominator and standardization target for all three models within a wave.

## S10B. Auxiliary equal-wave adjustment summaries

| **Sample** | **Model** | **Total records** | **Difference [95% CI], pp** | **Satterthwaite df** |
| --- | --- | --- | --- | --- |
| A30 | E0 | 71,218 | 12.49 [11.10, 13.88] | 161.06 |
| A30 | E1 | 71,218 | 5.99 [4.83, 7.16] | 147.47 |
| C30 | E0 | 64,000 | 12.66 [11.14, 14.17] | 159.05 |
| C30 | E1 | 64,000 | 5.98 [4.76, 7.20] | 138.07 |
| C30 | E2 | 64,000 | 5.31 [4.09, 6.54] | 139.86 |

Each of the nine waves has weight 1/9; variance assumes zero cross-wave covariance. Intervals use Satterthwaite t inference (Supplementary Table S11). Restricting A30 to C30 changes the equal-wave estimate by +0.16 percentage points for E0 and −0.01 for E1. Within C30, E1−E0 is −6.67 points and E2−E1 is −0.67 points. Changes are calculated before rounding and describe correlated estimates; they are not mediation or confounding-removed fractions. All 27 C30 models converged with full-rank matrices and no fitting warnings.

## S10C. Missingness among A30 records

| **Variable** | **Missing n** | **Unweighted missing %** |
| --- | --- | --- |
| Household income | 7,200 | 10.1 |
| Personal income | 5,114 | 7.2 |
| Marital status | 22 | 0.03 |
| Employment | 13 | 0.02 |
| Self-rated health | 46 | 0.06 |

The denominator is 71,218 A30 records. Missingness overlaps across variables; counts must not be added.

Pooled chained-equations sensitivity analysis used 50 imputations and ten iterations, including wave, official weight, exposure, outcome, core and extended covariates, and self-rated health. Household and personal income were imputed by predictive mean matching before conversion to within-wave ranks; categorical variables used categorical models. Exposure and outcome remained observed, and structural telephone non-asking was not imputed. Standardized differences were pooled using Rubin total variance and Barnard–Rubin finite-sample degrees of freedom, with Satterthwaite complete-data degrees of freedom (25, SR4).

## S10D. Pooled chained-equations estimates

| **Analysis** | **Records** | **Imputations** | **Difference [95% CI], pp** | **λ** | **Pooling df** |
| --- | --- | --- | --- | --- | --- |
| Extended adjustment | 71,218 | 50 | 5.38 [4.23, 6.54] | 0.003 | 144.59 |
| Health-conditional adjustment | 71,218 | 50 | 5.14 [3.98, 6.31] | 0.002 | 136.56 |

Health-conditional adjustment adds self-rated health as a five-category factor to the extended adjustment set. λ=B(1+1/m)/T is the missing-information proportion. These analyses assume conditional missing at random; conditioning on health does not identify a direct effect or mediation. Complete-case comparisons are in S10A–B.

A separate within-wave donor analysis failed convergence in 2010 before outcome-model fitting: household- and personal-income potential scale reduction factors were 1.213 and 1.264, exceeding the 1.10 threshold; autocorrelations were 0.391 and 0.444 (threshold 0.50). No effect estimate was obtained, and later waves were not fitted. The absence of an estimate is not a zero effect.

# Supplementary Table S11. Equal-wave summaries and model-specification sensitivity

The equal-wave index is Δ̂eq=(1/9)ΣΔ̂ₜ, with V̂eq=(1/81)ΣV̂ₜ under the working assumption of zero cross-wave covariance. Each V̂ₜ includes fitted-model and empirical-target uncertainty and their covariance. Normal-reference intervals use 1.96 SE; Satterthwaite t intervals use ν=(ΣV̂ₜ)²/Σ[V̂ₜ²/(Gₜ−1)], where Gₜ is the wave-specific province count. These are arithmetic summaries of nine different wave-specific empirical populations, not estimates for a fixed cross-year population.

The 36 specifications crossed three links (linear probability, logit and probit), three age forms (five-year groups, linear-plus-quadratic age and a four-degree-of-freedom natural spline), two education codings (five categories and the full valid factor), and presence or absence of an internet-by-sex term (24, SR3). Every specification used A30, official primary weights, the same core adjustment meaning, frequent minus nonfrequent use, and leisure exercise daily or several times per week. Predictions were standardized to each wave's weighted A30 population before equal-wave averaging. The specifications are correlated sensitivity analyses, not independent replications; no specification or wave was selected by statistical significance.

## S11A. Equal-wave association and the complete specification set

| **Quantity** | **Result** |
| --- | --- |
| A30 core model: equal-wave difference; SE | 5.99; 0.59 percentage points |
| A30 core model: normal-reference 95% interval | 4.84 to 7.15 percentage points |
| A30 core model: Satterthwaite t 95% interval | 4.83 to 7.16 percentage points |
| Specifications; wave-specific fits | 36; 324 |
| Specification-level equal-wave minimum; maximum | 5.43; 6.07 percentage points |
| Specification-level equal-wave median (interquartile range) | 5.80 (5.72–5.96) percentage points |
| Wave-by-specification minimum; maximum | 0.67; 10.28 percentage points |
| Negative equal-wave or wave-specific point estimates | 0 |

Positive point estimates do not imply that every wave-specific interval excludes zero. The two reference intervals apply to the same A30 core-model estimate. Identical-record adjustment summaries use Satterthwaite t intervals in S10B.

# Supplementary Table S12. Age, geographic scope, weighting, definitions and interview mode

Unless stated otherwise, these analyses retain core E1 adjustment, frequent minus nonfrequent general internet use and leisure exercise daily or several times per week. Each model is standardized to its analysis sample using the stated weights. Changes in age range, geographic scope, weighting, exposure, outcome or mode change the comparison being made; these estimates are not independent replications of the A30 comparison. Differences and intervals are in percentage points. S12A–B use equal-wave averages and normal-reference intervals; S12C is a single-wave analysis with province-proxy t intervals.

## S12A. Age, geographic scope and weighting comparisons

| **Analysis** | **Population or target** | **Equal-wave estimate, percentage points** | **95% interval, percentage points** | **Interpretation** |
| --- | --- | --- | --- | --- |
| Unweighted primary-population analysis | Primary roster | 6.13 | 5.20 to 7.07 | Omits official weights |
| Weight capping | Primary roster; occasion-specific 1st/99th percentiles | 6.09 | 4.94 to 7.24 | Alters the released weight distribution |
| Common-ten-province target | Ten provinces observed in all occasions; N=25,330 | 5.73 | 4.26 to 7.20 | Geographically restricted target, not national coverage |
| Age 18-or-older target | In-person respondents aged ≥18; N=90,059 | 5.52 | 4.35 to 6.69 | Support outside ages 30–74 is weaker in the exposure tails |
| Released weight field (weight), 2013–2021 | Five relevant occasions | 7.27 | 5.85 to 8.70 | Different released weight definition |
| Raking weight field (weight_raking), 2013–2021 | Same five occasions | 6.88 | 5.34 to 8.41 | Different released weight definition |

The ≥18 association sample requires observed outcome and complete adjustment covariates (90,059 records), unlike the outcome-independent D18 descriptive sample (90,457). The ten-province association sample contains 25,330 records and does not impose a common covariate distribution across years. Comparisons between the released weight and weight_raking fields cover only 2013, 2015, 2017, 2018 and 2021; other rows average nine waves. Unweighted targets use equal respondent weights, and capped-weight targets use capped weights.

## S12B. Alternative outcome and exposure definitions

| **Analysis** | **Equal-wave estimate, percentage points** | **95% interval, percentage points** | **Interpretation** |
| --- | --- | --- | --- |
| Daily leisure exercise | 3.27 | 2.30 to 4.25 | Nested outcome threshold |
| Leisure exercise daily or several times per week | 5.99 | 4.84 to 7.15 | Primary outcome threshold |
| Daily, several times/week, or several times/month leisure exercise | 8.66 | 7.37 to 9.95 | Nested outcome threshold |
| Any leisure exercise | 9.81 | 8.35 to 11.27 | Nested outcome threshold |
| Any versus never general internet use | 7.22 | 6.05 to 8.38 | Different exposure contrast and support |
| Frequent leisure-time internet use | 10.34 | 9.13 to 11.55 | Different internet-use construct |

The first four rows define nested A30 events using codes 1; 1–2; 1–3; and 1–4, respectively; increasing differences are not a dose–response series. Alternative exposure comparisons retain the primary A30 outcome but use their own exposure-eligible samples and corresponding standardization targets. Frequent leisure-time internet use is a distinct exposure with response direction harmonized before analysis.

## S12C. 2023 interview-mode and weight comparisons

| **Analysis** | **Population** | **Estimate, percentage points** | **95% interval, percentage points** | **Interpretation** |
| --- | --- | --- | --- | --- |
| 2023 in-person, weight1 | N=4,521 | 5.16 | −0.05 to 10.38 | Primary 2023 target |
| 2023 in-person, integrated weight2 | Same N=4,521 | 5.47 | 1.08 to 9.87 | Changes weight on the same roster |
| 2023 mixed mode, integrated weight2 with mode adjustment | N=4,782, including 261 telephone respondents | 5.52 | 1.45 to 9.59 | Different mode and target; does not establish mode invariance |

Each row uses its stated weight and empirical target. The mixed-mode sample adds 261 eligible telephone records to 4,521 in-person records. Structural telephone non-asking is not imputed. Intervals use the t reference with 15 degrees of freedom (16 province proxies). The primary weight1 interval includes zero; neither weight substitution nor mode adjustment establishes mode invariance.

# Supplementary Table S13. Survey-design information, finite-cluster inference and deletion checks

Official weights were available, but released geographic fields could not be verified as the official variance-estimation PSUs and strata. Province is therefore a geographic clustering proxy (22). Lower-level geographic fields were unavailable in 2017; a candidate stratum field was available only in 2010, without a verified variance-estimation role. Documentation did not establish that respondents or households never recurred across waves. Geographic-code overlaps cannot establish person or household recurrence, so zero cross-wave covariance remains a working assumption.

## S13A. Province counts and available design information

| **Year** | **Province proxies, G** | **t interval df** | **Lower geographic field** | **Official variance design** |
| --- | --- | --- | --- | --- |
| 2010 | 31 | 30 | s43 | Not recovered |
| 2011 | 26 | 25 | s43 | Not recovered |
| 2012 | 29 | 28 | s43 | Not recovered |
| 2013 | 28 | 27 | s45 | Not recovered |
| 2015 | 28 | 27 | s45 | Not recovered |
| 2017 | 28 | 27 | None released | Not recovered |
| 2018 | 28 | 27 | community_i | Not recovered |
| 2021 | 19 | 18 | community_i | Not recovered |
| 2023 | 16 | 15 | s45 | Not recovered |

The displayed G and G−1 apply to the A30 wave-specific models. Field availability alone does not verify its official sampling-unit role, nesting or compatibility with the analyzed mode and weights.

The finite-cluster sensitivity applied independent six-point multipliers to complete province influence totals within each wave, using 99,999 draws (SR5, SR6, SR7, SR8). Each draw perturbs influence contributions without refitting the full estimator. The full-estimator jackknife instead refitted the model and empirical standardization target after each of 233 wave-by-province deletions. These procedures assess uncertainty within the province-proxy framework and do not recover or validate the official multistage variance design.

## S13B. Finite-cluster interval comparisons

| **Analysis** | **Difference [95% interval], pp** | **SE, pp** |
| --- | --- | --- |
| Primary joint-influence reference | 5.99 [4.84, 7.15] | 0.59 |
| Independent-by-wave six-point finite-cluster multiplier | 5.99 [4.84, 7.16] | 0.59 |
| Full-estimator occasion-by-province deletion jackknife | 5.99 [4.79, 7.20] | 0.61 |

All rows summarize the same nine A30 binary point estimates with equal wave weights. The joint-influence reference uses a normal interval and zero cross-wave covariance; the multiplier and full-estimator jackknife rows use their respective uncertainty procedures.

## S13C. Leave-one-wave influence checks

| **Omitted year** | **Difference [normal 95% interval], pp** | **Shift, pp** |
| --- | --- | --- |
| 2010 | 6.48 [5.21, 7.74] | 0.48 |
| 2011 | 5.93 [4.71, 7.15] | −0.06 |
| 2012 | 6.02 [4.76, 7.28] | 0.03 |
| 2013 | 6.24 [4.98, 7.49] | 0.24 |
| 2015 | 5.89 [4.73, 7.05] | −0.11 |
| 2017 | 5.48 [4.21, 6.76] | −0.51 |
| 2018 | 5.70 [4.46, 6.93] | −0.29 |
| 2021 | 6.11 [4.88, 7.35] | 0.12 |
| 2023 | 6.10 [4.94, 7.26] | 0.10 |

Each row averages the other eight waves with weight 1/8; the shift is relative to the nine-wave index. Separately, geographic-province deletion removes a province from every wave in which it appears and refits the affected models and targets. Of 31 geographic-province deletions, Beijing gave the largest absolute change: the equal-wave difference was 6.32 percentage points, a shift of +0.33. Geographic deletion is an influence check, distinct from the wave-by-province jackknife variance calculation. These checks do not identify an event effect.

# Supplementary references

SR1. Davidov E, Muthen B, Schmidt P. Measurement invariance in cross-national studies: challenging traditional approaches and evaluating new ones. Sociol Methods Res. (2018) 47:631–636. doi: 10.1177/0049124118789708

SR2. Cernat A, Sakshaug JW. The impact of mixing survey modes on estimates of change: a quasi-experimental study. J Surv Stat Methodol. (2023) 11:1110–1132. doi: 10.1093/jssam/smac034

SR3. Simonsohn U, Simmons JP, Nelson LD. Specification curve analysis. Nat Hum Behav. (2020) 4:1208–1214. doi: 10.1038/s41562-020-0912-z

SR4. Barnard J, Rubin DB. Miscellanea. Small-sample degrees of freedom with multiple imputation. Biometrika. (1999) 86:948–955. doi: 10.1093/biomet/86.4.948

SR5. MacKinnon JG, Nielsen MØ, Webb MD. Fast and reliable jackknife and bootstrap methods for cluster-robust inference. J Appl Econom. (2023) 38:671–694. doi: 10.1002/jae.2969

SR6. MacKinnon JG, Webb MD. The wild bootstrap for few (treated) clusters. Econom J. (2018) 21:114–135. doi: 10.1111/ectj.12107

SR7. Cameron AC, Gelbach JB, Miller DL. Bootstrap-based improvements for inference with clustered errors. Rev Econ Stat. (2008) 90:414–427. doi: 10.1162/rest.90.3.414

SR8. Webb MD. Reworking wild bootstrap-based inference for clustered errors. Can J Econ. (2023) 56:839–858. doi: 10.1111/caje.12661

SR9. Ding P, VanderWeele TJ. Sensitivity Analysis Without Assumptions. Epidemiology. (2016) 27:368–377. doi:10.1097/EDE.0000000000000457.

SR10. VanderWeele TJ, Ding P. Sensitivity Analysis in Observational Research: Introducing the E-Value. Ann Intern Med. (2017) 167:268–274. doi:10.7326/M16-2607.
